# A Metabolomics and Molecular Networking Approach to Elucidate the Structures of Secondary Metabolites Produced by *Serratia marcescens* Strains

**DOI:** 10.3389/fchem.2021.633870

**Published:** 2021-03-16

**Authors:** Tanya Clements, Marina Rautenbach, Thando Ndlovu, Sehaam Khan, Wesaal Khan

**Affiliations:** ^1^ Department of Microbiology, Faculty of Science, Stellenbosch University, Stellenbosch, South Africa; ^2^ BioPep™ Peptide Group, Department of Biochemistry, Faculty of Science, Stellenbosch University, Stellenbosch, South Africa; ^3^ Faculty of Health Sciences, University of Johannesburg, Doornfontein, South Africa

**Keywords:** pigmented and non-pigmented *Serratia marcescens*, molecular networking, prodigiosin, serratamolide, glucosamine derivative, UPLC-MS^E^

## Abstract

An integrated approach that combines reverse-phase high-performance liquid chromatography (RP-HPLC), electrospray ionization mass spectrometry, untargeted ultra-performance liquid chromatography coupled to tandem mass spectrometry (UPLC-MS^E^) and molecular networking (using the Global Natural Products Social molecular network platform) was used to elucidate the metabolic profiles and chemical structures of the secondary metabolites produced by pigmented (P1) and non-pigmented (NP1) *Serratia marcescens* (*S. marcescens*) strains. Tandem mass spectrometry-based molecular networking guided the structural elucidation of 18 compounds for the P1 strain (including 6 serratamolides, 10 glucosamine derivatives, prodigiosin and serratiochelin A) and 15 compounds for the NP1 strain (including 8 serratamolides, 6 glucosamine derivatives and serratiochelin A) using the MS^E^ fragmentation profiles. The serratamolide homologues were comprised of a peptide moiety of two L-serine residues (cyclic or open-ring) linked to two fatty acid chains (lengths of C_10_, C_12_, or C_12:1_). Moreover, the putative structure of a novel open-ring serratamolide homologue was described. The glucosamine derivative homologues (i.e., *N*-butylglucosamine ester derivatives) consisted of four residues, including glucose/hexose, valine, a fatty acid chain (lengths of C_13_ – C_17_ and varying from saturated to unsaturated) and butyric acid. The putative structures of seven novel glucosamine derivative homologues and one glucosamine derivative congener (containing an oxo-hexanoic acid residue instead of a butyric acid residue) were described. Moreover, seven fractions collected during RP-HPLC, with major molecular ions corresponding to prodigiosin, serratamolides (A, B, and C), and glucosamine derivatives (A, C, and E), displayed antimicrobial activity against a clinical *Enterococcus faecalis* S1 strain using the disc diffusion assay. The minimum inhibitory and bactericidal concentration assays however, revealed that prodigiosin exhibited the greatest antimicrobial potency, followed by glucosamine derivative A and then the serratamolides (A, B, and C). These results provide crucial insight into the secondary metabolic profiles of pigmented and non-pigmented *S. marcescens* strains and confirms that *S. marcescens* strains are a promising natural source of novel antimicrobial metabolites.

## Introduction

**FIGURE 1:**
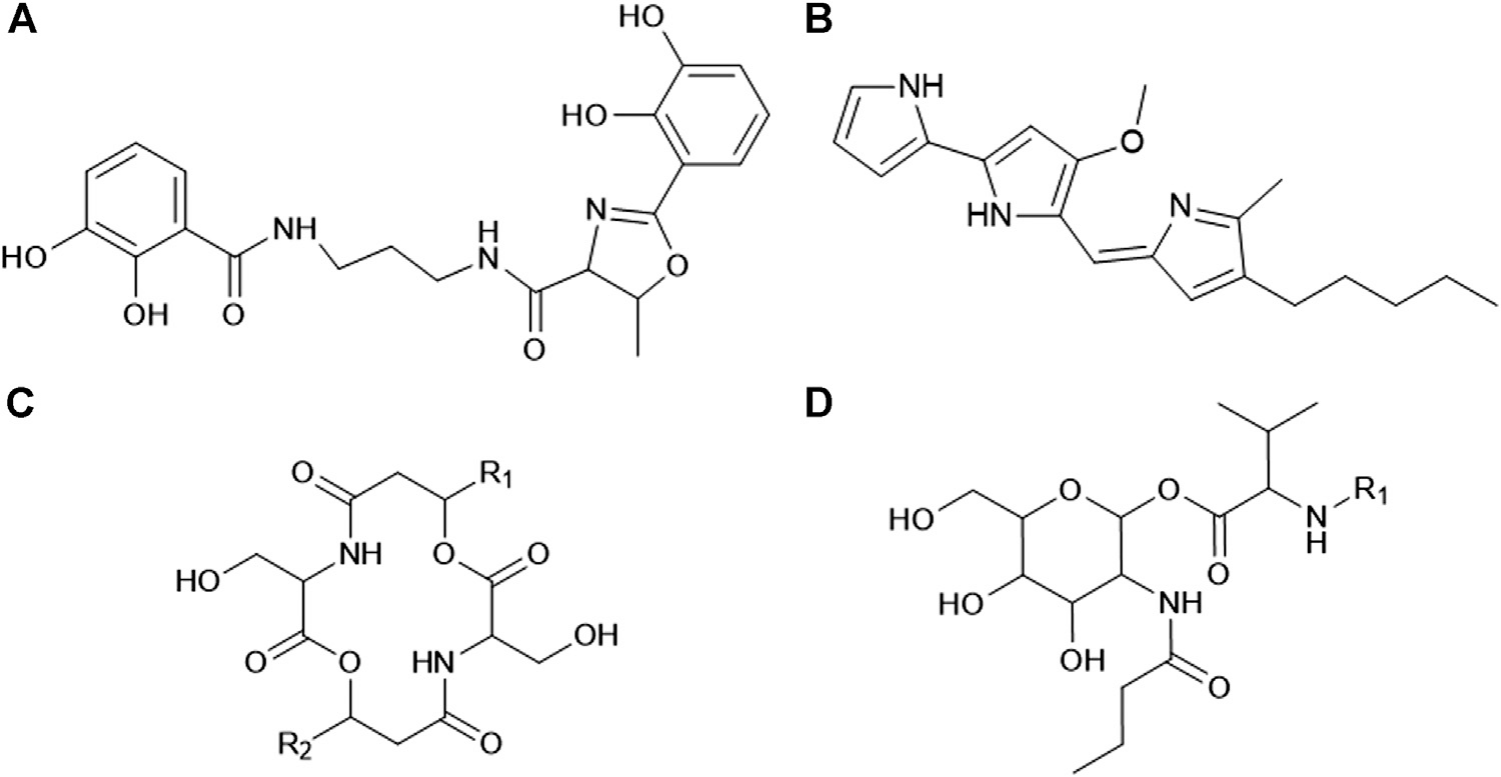
General structures of **(A)** serratiochelin A, **(B)** prodigiosin, **(C)** serratamolide and **(D)** glucosamine derivative (adapted from Dwivedi et al., 2008; Lee et al., 2011; Seyedsayamdost et al., 2012). The R_1_ and R_2_ groups on the serratamolides and glucosamine derivative structures indicate the fatty acyl moieties and these chains may vary in length and saturation (saturated, mono-unsaturated or poly-unsaturated).

*Serratia* species are facultatively anaerobic, Gram-negative rods that are classified into the Enterobacteriaceae family (Grimont and Grimont, 2015). The type strain of this genus, *Serratia marcescens* (*S. marcescens*), is widely known to produce a distinctive red pigment referred to as prodigiosin (Su et al., 2016). *Serratia plymuthica* (*S. plymuthica*), *Serratia rubidaea* (*S. rubidaea*), and *Serratia nematodiphila* (*S. nematodiphila*) have also been reported to synthesize this pigment, while non-pigmented strains of these species have been identified (Su et al., 2016). In recent years, pigmented and non-pigmented *Serratia* species have been recognized as a potential source of novel and structurally diverse bioactive secondary metabolites (Soenens and Imperial, 2019). These bioactive metabolites include the pigment prodigiosin as well as biosurfactants (lipopeptides and glycolipids), glucosamine derivatives, oocydin A, siderophores (such as serratiochelin A; Figure 1A), bacteriocins, carbapenem, althiomycin and serratin, among others (Clements et al., 2019a; Khilyas et al., 2019). Prodigiosin and serrawettins are two of the more extensively studied secondary metabolites due to their diverse biological activity and application as antitumor, antibacterial and antifungal agents (Stankovic et al., 2014; Eckelmann et al., 2018; Clements et al., 2019a).

Prodigiosin is a red, tripyrrole pigment (family of prodiginines) that is produced by several bacterial genera, such as *Serratia, Hahella, Streptomyces, Zooshikella, Vibrio,* and *Pseudomonas,* among others (Darshan and Manonmani, 2015). Prodigiosin has a general structure of 2-methyl-3-pentyl-6-methoxyprodiginine (Figure 1B) and is composed of three rings referred to as pyrrolic ring A, B, and C (Hage-Hülsmann et al., 2018; Yip et al., 2019). Research has indicated that it is synthesized via a bifurcated pathway encoded in a pigment (*pig*) gene cluster comprised of 14 genes (*pigABCDEFGHIJKLMN*) (Williamson et al., 2006). In contrast, serrawettins are non-ribosomally synthesized cyclodepsipeptides solely produced by several members of the *Serratia* genus. Serrawettins are classified as lipopeptides as they are composed of a hydrophilic peptide moiety and a hydrophobic fatty acid moiety. Three unique structures of serrawettins were initially identified, including serrawettin W1 (also referred to as serratamolide A), serrawettin W2 and serrawettin W3 (Wasserman et al., 1961; Matsuyama et al., 1985; Matsuyama et al., 1986; Matsuyama et al., 1992), while various analogues and homologues of serrawettin W1 and W2 have since been described (Dwivedi et al., 2008; Motley et al., 2016; Su et al., 2016). Serratamolide A and homologues of this lipopeptide (including serratamolide B to G) are comprised of a cyclic peptide moiety of two L-serine amino acids linked to two β-hydroxy fatty acid moieties that may vary in length (C_8_ to C_14_) of the fatty acyl chain and may be saturated or unsaturated (Figure 1C; Dwivedi et al., 2008). In comparison, serrawettin W2 and W3 are comprised of five amino acids linked to one β-hydroxy fatty acid (Matsuyama et al., 1986; Matsuyama et al., 1992).

Glucosamine derivatives (also referred to as *N*-butylglucosamine ester derivatives) are non-ribosomally synthesized diacylated peptoglucosamine derivatives (Dwivedi et al., 2008; Khilyas et al., 2019) produced by *Serratia* species. They have not been extensively studied and to date only Dwivedi et al. (2008) characterized glucosamine derivatives produced by a pigmented *S. marcescens* SHHRE645 strain. The identified glucosamine derivative homologues (A to C) were comprised of four residues, including glucose, valine, butyric acid and a saturated or unsaturated β-fatty acid residue of varying length (Figure 1D). It was observed that the glucosamine derivatives were co-produced with serratamolide homologues. Although extensive research into the biological properties of glucosamine derivative homologues (A to C) is still required, Dwivedi et al. (2008) reported on the anti-mycobacterial activity for each homologue.

To date, several analytical methods have been utilized to elucidate the detailed structures and composition of secondary metabolites produced by *Serratia* species, including mass spectrometry (MS), liquid chromatography (LC), gas chromatography (GC), and nuclear magnetic resonance (NMR) (Dwivedi et al., 2008; Thies et al., 2014; Eckelmann et al., 2018). Chromatography methods are commonly coupled with MS for the reliable separation and identification of bioactive compounds (Fu et al., 2019). Moreover, tandem MS analysis allows for the detection and structural characterization of a broad range of compounds and distinguishes between closely related forms of the same compound produced by a bacterial strain (Knolhoff et al., 2015). For example, a study by Eckelmann et al. (2018) unraveled the secondary metabolic profile of a pigmented *S. marcescens* strain MSRBB2 using high performance LC (HPLC) linked to high resolution MS (HRMS) and HRMS^n^ analysis. The characteristic fragmentation patterns produced by HRMS^n^ analysis allowed for the identification and elucidation of 33 compounds, including 7 prodiginines and 26 serratamolides (Eckelmann et al., 2018).

Recently, a novel approach has been developed to analyze large data sets from tandem MS spectra of natural product extracts. This approach involves the Global Natural Products Social (GNPS) molecular network platform (http://gnps.ucsd.edu) as an open-access tool that employs a computational algorithm to compare the degree of similarity between tandem MS spectra and generates a molecular network comprised of clusters of structurally related metabolites. Thus, a visual representation of the structural relationships between natural compounds within crude extracts is generated, where a node within the network represents a compound and the relatedness between two compounds (nodes) is referred to as an edge. Moreover, GNPS allows for the comparison of the tandem MS spectra to the publicly available spectral library for rapid identification of known compounds (Wang et al., 2016; Philippus et al., 2018).

Here, we present an integrated approach to identify and structurally elucidate the naturally produced complex of secondary metabolites of a pigmented (P1) and non-pigmented (NP1) *S. marcescens* strain using reverse-phase high performance liquid chromatography (RP-HPLC), electrospray ionization mass spectrometry (ESI-MS), untargeted ultra-performance liquid chromatography coupled to tandem mass spectrometry (UPLC-MS^E^) and molecular networking (using the GNPS molecular network platform) analysis. In addition, the susceptibility of a clinical *Enterococcus faecalis* (*E. faecalis*) strain to fractions collected during RP-HPLC analysis was evaluated using disc diffusion assays (all fractions) and broth microdilution assays to determine the minimum inhibitory concentration (MIC) and minimum bactericidal concentration (MBC) of selected fractions.

## Materials and Methods

### Bacterial Strains

A previous study by Clements et al. (2019b) outlined the isolation of the P1 and NP1 *S. marcescens* strains from an oil refinery effluent sample and a river water sample, respectively as well as the molecular identification of the strains. The two *S. marcescens* strains were streaked from the glycerol stocks onto Nutrient agar (Merck, Johannesburg, South Africa) and were incubated at 30°C for 18–24 h. The *S. marcescens* P1 and NP1 strains were deposited in the South African Rhizobium Culture Collection (SARCC no. for P1: 3059; SARCC no. for NP1: 3060). The clinical *E. faecalis* S1 strain, used for the disc diffusion and MIC assays, was streaked from the glycerol stock onto tryptone soy agar (Biolab, Merck, Johannesburg, South Africa) supplemented with 6 g/L yeast extract (Biolab, Merck, Johannesburg, South Africa) (TSAYE_0.6%_) and was incubated at 37°C for 18–24 h. The clinical *E. faecalis* S1 strain is curated and accessible in the Water Resource Laboratory culture collection in the Department of Microbiology at Stellenbosch University.

### Production and Extraction of Secondary Metabolites

The production and partial purification of secondary metabolites was performed as described by Clements et al. (2019b), with slight modifications to the scale of production (increased volume) and the use of peptone powder in culture media (Biolab, Merck, Johannesburg, South Africa). Briefly, seed cultures were grown overnight by inoculating the *S. marcescens* P1 and NP1 strains into 10 ml Luria Bertani broth (Biolab, Merck, South Africa) in triplicate and incubating on a test tube rotator (MRCLAB, London, United Kingdom) at 30°C. Each seed culture was subsequently inoculated into a 2 L baffled flask containing 500 ml Peptone Glycerol (PG, pH 7.2 ± 0.2) broth [composed of 5 g peptone powder and 10 ml glycerol (Promega, Wisconsin, United States) in 1 L distilled water] in triplicate, which was incubated on an orbital shaker (MRCLAB, London, United Kingdom) at 30°C for 120 h at 120 rpm. Following a 5 days incubation period, the P1 and NP1 broth cultures were centrifuged at 10,000 rpm for 20 min at 4°C to obtain the cell free supernatant. The cell free supernatants were lyophilized and dissolved in 70% HPLC-grade acetonitrile (Romil, Darmstadt, Germany) in analytical quality water (milliQ water) (*v/v*). The acetonitrile soluble fraction was transferred into a sterile McCartney bottle and lyophilized. This step was repeated thrice to further purify the crude extracts. The triplicate crude extracts were then pooled, lyophilized, analytically weighed and stored at −20°C until further use.

### Purification and Detection of Secondary Metabolites

The P1 and NP1 crude extracts were subjected to RP-HPLC (Finnigan Survey UV-VIS Plus detector, Thermo-Scientific, Waltham, MA, United States) analysis, in the Department of Microbiology, in order to obtain purified fractions. The lyophilized P1 and NP1 crude extracts were dissolved in 40% acetonitrile in milliQ water (*v/v*) to a concentration of 10.00 mg/ml and were injected into a Discovery BIO Wide Pore C_18_ HPLC column (10 μm, 250 × 10 mm; Sigma-Aldrich, St. Louis, United States). Liquid chromatography was conducted with milliQ water containing 0.1% trifluoroacetic acid (TFA; *v/v*; Sigma-Aldrich, St. Louis, United States) as solution A and acetonitrile containing 0.1% TFA (*v/v*) as solution B. An isocratic flow at 40% B from 0 to 2 min for sample loading was followed by a linear gradient from 40 to 95% B from 2 to 36 min. The column was washed for 2 min with 95% B and then regenerated with a reversed gradient from 95 to 40% B from 38 to 45 min. Chromatography of compounds was followed by continuous monitoring of the absorbance at 230 and 254 nm.

The absorbing peak fractions collected from the RP-HPLC were analyzed using a Waters Synapt G2 high resolution mass spectrometer fitted with a Z spray electrospray ionization source (Waters Corporation, Milford, United States) at the LCMS unit at the Central Analytical Facility (CAF, Stellenbosch University, South Africa). For direct mass analysis, 3 µl of the sample [250 μg/ml; dissolved in 50% acetonitrile in milliQ water (*v/v*)] was injected into the ionization source at a flow rate of 0.3 ml/min. The analytes were subjected to a capillary voltage of 2.5 kV, cone voltage of 15 V, a source temperature of 120°C, desolvation gas (N_2_) flow of 650 L/h and desolvation temperature at 275°C. Data acquisition in the positive mode was performed by MS scanning a second analyser through the mass to charge (*m/z*) range of 300–1,500 in centroid mode. The high resolution mass calibration was done with sodium formate and in-analysis single point lock spray calibration using leucine enkephalin (*m/z* = 556.2771). The ESI-MS data was processed using MassLynx software version 4.1 (Waters Corporation, Milford, United States). The accurate masses and molecular formula of the detected compounds were used to search online databases, such as Norine (https://bioinfo.lifl.fr/norine/) and PubChem (https://pubchem.ncbi.nlm.nih.gov/), of known natural products and an extensive literature search was conducted for the putative identification of the metabolites.

### Analysis Utilizing Untargeted Ultra-performance Liquid Chromatography Linked to Tandem Mass Spectrometry

In order to elucidate the structure of each of the detected compounds, all fractions were subjected to untargeted ultra-performance liquid chromatography coupled to tandem mass spectrometry (UPLC-MS^E^) analysis at the LCMS unit at the Central Analytical Facility (CAF, Stellenbosch University). The RP-HPLC purified fractions obtained from the P1 and NP1 crude extracts were prepared in 70% acetonitrile in milliQ water (*v/v*) to a concentration of 250 μg/ml and were subjected to the Waters Synapt G2 high resolution mass spectrometer linked to an Acquity UPLC™ for UPLC-MS analysis. Three microlitres of each fraction (1.00 mg/ml) was separated on an UPLC C_18_ reverse-phase analytical column (Acquity UPLC^®^ HSS T3, 1.8 μm particle size, 2.1 × 150 mm, Waters Corporation, Dublin, Ireland). The compound separation via UPLC was facilitated with milliQ water containing 0.1% (*v/v*) formic acid as solution A and acetonitrile containing 0.1% (*v/v*) formic acid as solution B. The gradient was developed at a flow rate of 0.300 ml/min as follows: 60% A from 0 to 0.5 min for loading, linear gradient from 20 to 80% (B) from 0.5 to 14 min and 0–100% (B) from 14 to 15 min. The ESI-MS instrument settings for monitoring the chromatography were as described for the direct mass analysis.

High resolution collisionally induced dissociation (CID) analysis was conducted in the MS^E^ mode (MS/MS) during the UPLC-MS analysis and was monitored on a second MS channel. The CID was conducted at a collision energy gradient of 20–70 eVat 1  s MS/MS scan time. Data was collected in the second mass analyser (MS2) through *m/z* range of 40–1,500 in centroid mode. The rest of the instrument settings were as described above. The UPLC-MS^E^ data was processed using Mass Lynx software version 4.1 (Waters Corporation).

### Molecular Networking Analysis

The Waters RAW files for the P1 and NP1 fractions obtained after UPLC-MS^E^ analysis were converted into an Analysis Base File (ABF) format using Reifycs Analysis Base File Converter before data processing. Ion chromatogram extraction, alignment and peak deconvolution of the ABF converted files was then conducted using MS-DIAL software version 4.24. The aligned results were exported as a mascot generic format (mgf) file for P1 and NP1 (Tsugawa et al., 2015). Thereafter, the mgf files for the two strains were uploaded to the GNPS platform (http://gnps.ucsd.edu) and a molecular network was created using the workflow published by Wang et al. (2016). Briefly, the data was filtered by removing all MS/MS fragment ions within +/−17 Da of the precursor *m/z*. The MS/MS spectra were window filtered by selecting only the top six fragment ions in the +/−50 Da window throughout the spectrum. The following parameters were then used in the workflow: precursor ion mass tolerance was set to 0.03 Da, MS/MS fragment ion tolerance of 0.02 Da, cosine score above 0.6 and minimum matched peaks of ten. Further, edges between two nodes were kept in the network only if each of the nodes appeared in each other’s respective top 10 most similar nodes. The spectra in the network were then searched against GNPS’ spectral libraries. All matches retained between network spectra and library spectra were required to have a score above 0.7 and at least six matched peaks (Wang et al., 2016). The output of the molecular network was visualized using Cytoscape version 3.8.0. The nodes (compounds) originating from media and solvent controls (acetonitrile) were excluded from the original network in order to visualize the secondary metabolites derived from the P1 and NP1 strains.

### Antimicrobial Susceptibility Testing

#### Disc Diffusion Assay

The lyophilized fraction stocks (obtained after RP-HPLC) were dissolved in 100% dimethyl sulfoxide (DMSO; Sigma-Aldrich, St. Louis, United States) to a concentration of 20.00 mg/ml (stock concentration). Following solubility of each fraction, an aliquot of each fraction was diluted in milliQ water to a final concentration of 3.00 mg/ml (with a final DMSO concentration of 15%). These aliquots (at 3.00 mg/ml) were then subjected to antimicrobial testing using a standard disc diffusion assay as described by Das et al. (2008) and Ndlovu et al. (2017). Briefly, the *E. faecalis* S1 test strain was inoculated into 5 ml of tryptone soy broth (Biolab, Merck, Johannesburg, South Africa) supplemented with 6 g/L yeast extract (TSBYE_0.6%_) and was incubated at 37°C for 18–24 h. Following incubation, 100 µl of the *E. faecalis* S1 suspension was spread plated onto TSAYE_0.6%_ to create a microbial lawn. Thereafter, 6 mm filter paper discs (Oxoid, Basingstoke, United Kingdom) were placed onto the microbial lawn using a sterile needle and 50 µl of each fraction (3.00 mg/ml in 15% DMSO) was pipetted directly onto the filter paper (in triplicate) in order to create an antimicrobial disc. A negative control of 50 µl of 15% (*v/v*) DMSO was included and the plates were incubated for 24 h at 37°C.

#### Minimum Inhibitory and Bactericidal Concentration

Fractions were selected for further antimicrobial testing based on the results of the disc diffusion assay and the quantity of each fraction remaining. The fraction stocks were prepared in 100% DMSO (as outlined in 2.6.1) to 20.00 mg/ml and aliquots were prepared and diluted to 6.00 mg/ml (DMSO concentration of 30%) using milliQ water. A serial dilution of the fractions at 6.00 mg/ml was then conducted using 30% DMSO to obtain a concentration range of 1.50–6.00 mg/ml (DMSO concentration of 30%). The selected fractions (at various concentrations) were subjected to antimicrobial testing using a broth microdilution susceptibility assay as outlined by the European Committee on Antimicrobial Susceptibility Testing (European Communitee on Antimicrobial Susceptibility Testing, 2018) and Magalhães and Nitschke et al. (2013). Briefly, *E. faecalis* S1 was inoculated into 5 ml of TSBYE_0.6%_ and was incubated at 37°C for 18–24 h. Following overnight incubation, the culture was diluted to an optical density (OD) of approximately 0.08 at 625 nm corresponding to ∼10^7^ colony forming units (CFU)/ml. One hundred microlitres of each fraction (at the various concentrations) was dispensed into the respective wells of a Greiner CELLSTAR^®^ 96-well culture plate (Merck, Johannesburg, South Africa) containing 100 μl of sterile TSBYE_0.6%_ (resulting in a final test concentration of 0.75–3.00 mg/ml in 15% DMSO). All the wells of the microtiter plate were inoculated with 10 μl of the bacterial inoculum (final concentration of ∼10^5^ CFU/ml). Sterile broth, 15% DMSO and the OD adjusted inoculum were included in the assay as positive controls, while sterile broth with 15% DMSO was included as a negative control. All the tests were performed in triplicate and the microtiter plate was incubated for 18–24 h at 37°C. Following incubation, the absorbance was measured using a microtiter plate reader. The MIC was assigned as the lowest concentration of the respective compounds that reduced bacterial numbers by ≥90% (Yasir et al., 2019). The MBC was determined as previously described by Das et al. (2008). Briefly, after the MIC determination, the wells that showed ≥90% inhibition of growth were identified and 20 µl of each well was spot plated onto TSAYE_0.6%_ *.* The lowest concentration showing no revival of the test culture was recorded as the MBC.

## Results

### Purification and Identification of Secondary Metabolites

Reverse-phase HPLC was used to fractionate the secondary metabolites within the P1 and NP1 crude extracts. The RP-HPLC analysis of the P1 and NP1 crude extracts revealed *n* = 11 absorption peaks in the P1 crude extract and *n* = 8 absorption peaks in the NP1 crude extract, between 8 and 35 min. All the collected fractions were then subjected to ESI-MS analysis to detect potential metabolites produced by each strain. As a result, a combined total of 21 compounds, produced by the P1 and/or NP1 strains, were detected [numbered as compounds **1**–**21** and arranged according to the retention time (Rt), Supplementary Table S1. The positive mode ESI-MS analysis of each collected fraction obtained from the P1 crude extract revealed a profile with major molecular ions at *m/z* 430.1609, 324.2073, 515.3331, 557.3804, 541.3485, 543.3644, 571.3931, and 559.3953 (compounds **1**, **2**, **4**, **7**, **9**, **13**, **18**, and **14**, respectively). The ESI-MS analysis of the NP1 crude extract revealed a similar profile with the major molecular ions of *m/z* 430.1609, 515.3331, 557.3804, 541.3485, 543.3644, 559.3953, and 585.4117 (compounds **1**, **4**, **7**, **9**, **13**, **14**, and **16**, respectively). The corresponding sodium and potassium adducts of the compounds from the P1 and NP1 fractions were also detected (Supplementary Table S1). It should be noted that several compounds co-eluted with the major compounds (higher molecular ion signal) (Supplementary Table S1). Due to the co-elution of presumptive known (*n* = 12) and unknown (*n* = 9) compounds detected in the P1 and/or NP1 crude extracts, UPLC-MS^E^ coupled with molecular networking analysis was used to cluster structurally similar compounds and elucidate the putative structures of the unknown compounds and facilitate the confirmation of the known structures.

### Molecular Networking Analysis

**FIGURE 2:**
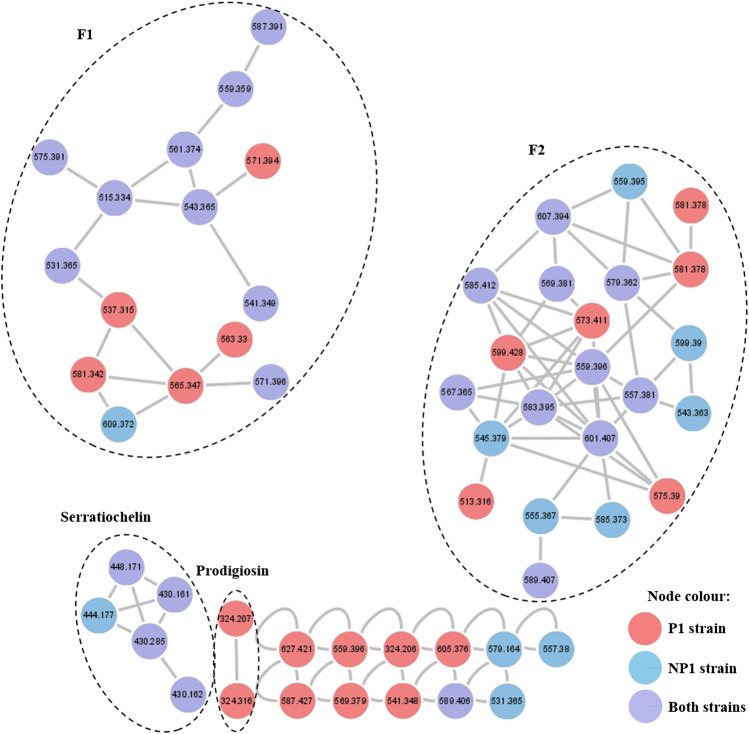
Molecular network of the secondary metabolites produced by the *S. marcescens* P1 and NP1 strains generated using the UPLC-MS^E^ data. Nodes are labeled with the corresponding *m/z* values (detected in the positive mode) and respective strain colors are indicated in the node color key. The four clusters are labeled with either the corresponding compound family name detected using GNPS or MS-DIAL library search, or F1 and F2 corresponding to metabolite clusters that were not identified using the library searches.

**FIGURE 3:**
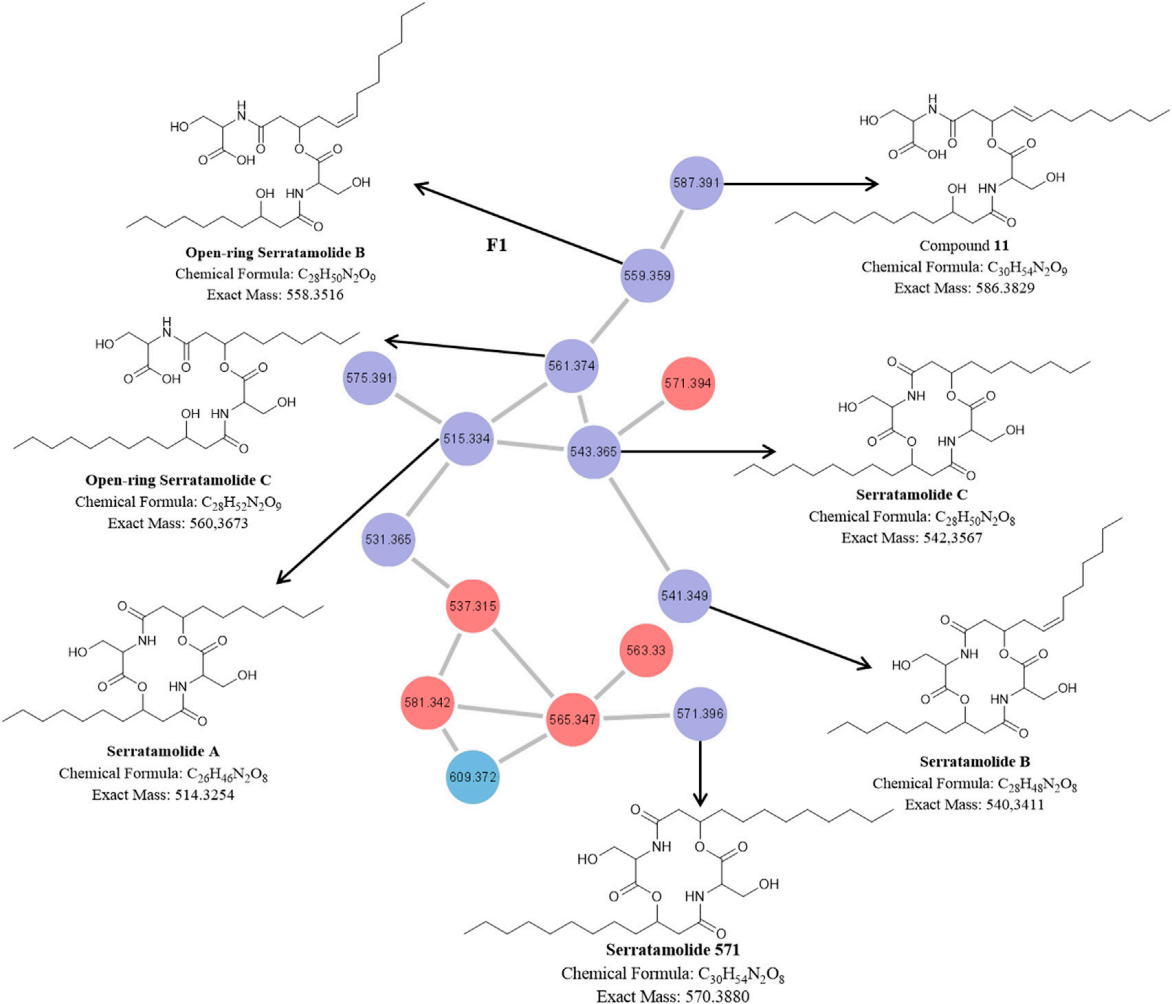
The molecular network of cluster F1 (magnified) with the corresponding structures of known and unknown serratamolide homologues [previously identified by and adopted from Dwivedi et al. (2008) and Eckelmann et al. (2018)] detected in the P1 and NP1 crude extracts identified using UPLC-MS^E^ analysis. Red nodes = metabolites produced by the P1 strain; Blue nodes = metabolites produced by the NP1 strain; Purple nodes = metabolites produced by both P1 and NP1 strains.

The fractions obtained after RP-HPLC of the secondary metabolites extracted from the P1 and NP1 strains were subjected to UPLC-MS^E^ analysis and the raw MS^E^ data was used to generate a molecular network. Analysis of the molecular network revealed four clusters (Figures 2, 3, 5), including families of serratiochelin (comprised of five nodes) and prodigiosin (comprised of two nodes) that were identified using the GNPS and MS-DIAL libraries, respectively, and two unidentified F1 (comprised of 15 nodes) and F2 (comprised of 22 nodes) families that did not correspond to compounds within the GNPS and MS-DIAL libraries. In general, the majority of the metabolites (nodes) clustered in the F1, F2 and serratiochelin families and were detected in both P1 and NP1 strains; however, only the P1 strain produced compounds in the prodigiosin cluster. The entire network was formed by 55 nodes, including 11 individual nodes.

### UPLC-MS^E^ Analysis

Following molecular networking, the structural elucidation of the four distinct metabolite clusters produced by the *S. marcescens* P1 and NP1 strains was conducted by analyzing the fragmentation profiles of the UPLC-MS^E^ data. The identified serratiochelin cluster (Figure 2) revealed a node with *m/z* 430.1609 [C_21_H_23_N_3_O_7_ + H]^+^ (compound **1**) corresponding to the peak observed at 3.76 min in the UPLC-MS^E^ data for the P1 and NP1 strains (Table 1; Supplementary Figure S1). The fragmentation pattern of **1**, which was observed using UPLC-MS^E^, corresponded to the serratiochelin A (also referred to as serranticin) structure (Kuo et al., 2011; Seyedsayamdost et al., 2012). The prodigiosin cluster (Figure 2) revealed a node with *m/z* 324.2073 [C_20_H_25_N_3_O+ H]^+^ (compound **2**) corresponding to the peak observed at 8.48 min in the UPLC-MS^E^ data for the P1 strain (Table 1; Supplementary Figure S2). The fragmentation pattern of **2**, which was observed using UPLC-MS^E^, corresponded to the prodigiosin structure (Lee et al., 2011; Su et al., 2016). The fragmentation profiles and structural elucidation of the compounds detected in clusters F1 and F2 will be discussed according to each metabolite family (cluster). A summary of the UPLC-MS^E^ data for the metabolites within these clusters produced by the P1 and NP1 strains is presented in Tables 1, 2 and Supplementary Figures S22, S23, and the structures of the known metabolites and putative structures of the unknown compounds produced by the P1 and NP1 strains are depicted in Figures 3, 5 and Supplementary Figures S3–S21.

**TABLE 1:** Summary of the serratamolide homologues, prodigiosin and serratiochelin A detected in the crude extracts obtained from *S. marcescens* P1 and NP1 that were identified using UPLC-MS^E^ analysis.

Crude extract	UPLC Rt (min)	Compound no	Proposed compound identity	Proposed fatty acid chain lengths	^d^Molecular formula	*m/z* [M + H]^+^	^a^Experimental *M*_*r*_	^b^Theoretical *M*_*r*_	^c^Mass error (∆_ppm_)	Major UPLC-MS^E^ fragments
P1, NP1	3.76	**1**	Serranticin/Serratiochelin A	N/A	C_21_H_23_N_3_O_7_	430.1609	429.1530	429.1536	1.49	137, 192, 194, 211, 220, 250, 277, 294
P1	8.48	**2**	Prodigiosin	N/A	C_20_H_25_N_3_O	324.2073	323.1994	323.1998	1.36	252, 266, 292, 309
P1, NP1	11.20	**3**	Open-ring serratamolide B	C_10_ + C_12:1_	C_28_H_50_N_2_O_9_	559.3600	558.3522	558.3516	-1.03	258, 266, 276, 284, 302, 541
P1, NP1	11.28	**6**	Open-ring serratamolide C	C_10_ + C_12_	C_28_H_52_N_2_O_9_	561.3749	560.3671	560.3673	0.89	212, 240, 258, 268, 276, 286, 304, 543
P1, NP1	11.49	**4**	Serratamolide A/Serrawettin W1	C_10_ + C_10_	C_26_H_46_N_2_O_8_	515.3331	514.3252	514.3254	0.47	212, 240, 258, 276, 469, 487, 497
NP1	12.46	**8**	^e^Open-ring serratamolide 585	C_12:1_ + C_12:1_	C_30_H_52_N_2_O_9_	585.3738	584.3660	584.3673	2.26	238, 266, 284, 302, 567
P1, NP1	12.77	**9**	Serratamolide B	C_10_ + C_12:1_	C_28_H_48_N_2_O_8_	541.3485	540.3404	540.3411	0.55	212, 240, 258, 266, 276, 284, 302, 513, 523
P1, NP1	13.06	**13**	Serratamolide C	C_10_ + C_12_	C_28_H_50_N_2_O_8_	543.3644	542.3565	542.3567	0.44	212, 240, 258, 268, 276, 286, 304, 515, 525
NP1	13.76	**11**	Open-ring serratamolide 587	C_12_ + C_12:1_	C_30_H_54_N_2_O_9_	587.3908	586.3830	586.3829	-0.13	266, 268, 284, 286, 302, 304, 569
P1, NP1	14.02	**18**	Serratamolide 571	C_12_ + C_12_	C_30_H_54_N_2_O_8_	571.3931	570.3852	570.3880	4.98	222, 240, 268, 286, 304, 525, 553

^a^ Experimental monoisotopic *M*_*r*_ of compound was calculated using the Time-of-Flight (TOF) transform function in the MassLynx 4.1 software package.

^b^ Theoretical monoisotopic *M*_*r*_ of compound was calculated using ChemDraw Ultra 12.0 software package.

^c^ Mass error in ppm = $\left(\frac{\text{Theoretical}\;M_{r}\;\;-\;\text{Experimental}\;M_{r}\;}{\text{ Theoretical}\;M_{r}}\right)\times 10^{6}$

^d^ Theoretical molecular formula of compound was calculated using ChemDraw Ultra 12.0 software package and experimental molecular formula was confirmed using the MassLynx 4.1 software package.

^e^ Novel serratamolide homologue.

N/A, not applicable.

Bold entries correspond to compound numbers.

**TABLE 2:** Summary of the glucosamine derivative homologues detected in the crude extracts obtained from *S. marcescens* P1 and NP1 that were identified using UPLC-MS^E^.

Crude extract	UPLC Rt (min)	Compound no	Proposed compound identity	Fatty acid chain length	^d^Molecular formula	*m/z* [M + H]^+^	^a^Experimental *M*_*r*_	^b^Theoretical *M*_*r*_	^c^Mass error (∆_ppm_)	Major UPLC-MS^E^ fragments
P1	11.44	**5**	^e^Glucosamine derivative D	C_14_	C_29_H_54_N_2_O_9_	575.3902	574.3828	574.3829	1.11	196, 214, 232, 326, 344, 366
P1, NP1	11.98	**7**	^e^Glucosamine derivative E	C_14:1_	C_29_H_52_N_2_O_8_	557.3804	556.3722	556.3724	-0.31	196, 214, 232, 326, 348
NP1	12.67	**10**	^e^Glucosamine derivative F	C_13_	C_28_H_52_N_2_O_8_	545.3787	544.3719	544.3724	3.01	196, 214, 232, 314, 336
P1, NP1	12.94	**12**	^e^Glucosamine derivative G	C_15_	C_30_H_56_N_2_O_9_	589.4056	588.3985	588.3986	1.60	196, 214, 232, 340, 358, 380
P1, NP1	12.95	**14**	Glucosamine derivative C	C_14_	C_29_H_54_N_2_O_8_	559.3953	558.3877	558.3880	1.15	196, 214, 232, 328, 350
P1, NP1	13.14	**15**	^e^Glucosamine derivative H	C_16:2_	C_31_H_54_N_2_O_8_	583.3947	582.3868	582.3880	2.13	196, 214, 232, 352, 374
P1, NP1	13.50	**16**	Glucosamine derivative A	C_16:1_	C_31_H_56_N_2_O_8_	585.4117	584.4035	584.4037	-0.10	196, 214, 232, 354, 376
P1	15.69	**19**	Glucosamine derivative B	C_15_	C_30_H_56_N_2_O_8_	573.4136	572.4034	572.4037	-3.42	196, 214, 232, 342, 364
P1	16.01	**17**	^e^Glucosamine derivative I	C_17:1_	C_32_H_58_N_2_O_8_	599.4265	598.4203	598.4193	1.24	196, 214, 232, 368, 390
P1	16.28	**20**	^e^Glucosamine derivative J	C_16:1_	C_33_H_58_N_2_O_9_	627.4192	626.4114	626.4142	4.69	256, 274, 296, 354, 376
P1	16.50	**21**	^e^Glucosamine derivative K	C_16_	C_31_H_58_N_2_O_8_	587.4268	586.4187	586.4193	0.55	196, 214, 232, 356, 378

^a^ Experimental monoisotopic *M*_*r*_ of compound was calculated using the TOF transform function in the MassLynx 4.1 software package.

^b^ Theoretical monoisotopic *M*_*r*_ of compound was calculated using ChemDraw Ultra 12.0 software package.

^c^ Mass error in parts per million (ppm) = $\left(\frac{\text{Theoretical}\;M_{r}\;\;-\;\text{Experimental}\;M_{r}\;}{\text{ Theoretical}\;M_{r}}\right)\times 10^{6}$

^d^ Theoretical molecular formula of compound was calculated using ChemDraw Ultra 12.0 software package and experimental molecular formula was confirmed using the MassLynx 4.1 software package.

^e^ Novel glucosamine derivative homologues.

Bold entries correspond to compound numbers.

#### Structural Elucidation of Serratamolide Homologues

The molecular network of the F1 cluster and corresponding structures for the known and novel compounds in the F1 cluster are presented in Figure 3. The F1 cluster (Figure 3) revealed nodes with *m/z* 515.3331 [C_26_H_46_N_2_O_8_ + H]^+^ (compound **4**), 541.3485 [C_28_H_48_N_2_O_8_ + H]^+^ (compound **9**), 543.3644 [C_28_H_50_N_2_O_8_ + H]^+^ (compound **13**), 561.3749 [C_28_H_52_N_2_O_9_ + H]^+^ (compound **6**), 559.3600 [C_28_H_50_N_2_O_9_ + H]^+^ (compound **3**), and 571.3931 [C_30_H_54_N_2_O_8_ + H]^+^ (compound **18**), which corresponded to peaks observed in the UPLC-MS^E^ data for the P1 and NP1 strains (Table 1; Supplementary Figures S3–S8, S22). In addition, the F1 cluster (Figure 3) revealed a node with *m/z* 587.3908 [C_30_H_54_N_2_O_9_ + H]^+^ (compound **11**) corresponding to the peak observed at 13.76 min in the UPLC-MS^E^ data for the NP1 strain (Table 1; Supplementary Figures S9, S22). The fragmentation profiles of these seven compounds (**3**, **4**, **6**, **9**, **11**, **13**, and **18**) correspond to cyclic or open-ring serratamolide homologues that have previously been reported (Dwivedi et al., 2008; Eckelmann et al., 2018).

Compound **4** was thus comprised of two serine residues (cyclic peptide moiety) coupled to two saturated hydroxydecanoic acyl groups (C_10_) (Figure 3) confirming the identity of the compound as serratamolide A (Dwivedi et al., 2008). In addition, **18** was comprised of two serine residues (cyclic peptide moiety) linked to two saturated hydroxydodecanoic acyl groups (C_12_), which corresponded to a serratamolide homologue reported by Eckelmann et al. (2018) (Figure 3). In contrast, **9** and **13** were comprised of two serine residues (cyclic peptide moiety) coupled to a 3-hydroxydecanoic acyl (C_10_) and a hydroxydodecanoic acyl (C_12_) (Figure 3). Compound **13** thus corresponded to the previously described serratamolide C (Dwivedi et al., 2008). However, compound **9** had a double bond in the hydroxydodecanoic acyl (C_12:1_) residue (Figure 3) confirming the structure as serratamolide B (Dwivedi et al., 2008). The double bond position was putatively placed at the C_5_ position (from the C=O group) of the fatty acyl chain, as this corresponded to the position previously reported in literature (Eckelmann et al., 2018). Compounds **3** and **6** had identical fatty acyl chain lengths as serratamolide B and C, respectively, however, the addition of a hydroxyl group, based on the experimental molecular formula (elemental composition) predicted using the MassLynx 4.1 software package, in the compound suggested that both lipopeptides were open-ring structures and corresponded to the fragmentation pattern of the previously described open-ring serratamolides B and C (Eckelmann et al., 2018) (Figure 3). Similarly, **11** was comprised of one serine residue coupled to one saturated hydroxydodecanoic acyl (C_12_) and one serine residue coupled to one unsaturated hydroxydodecanoic acyl (C_12:1_). However, the addition of a hydroxyl group [based on the experimental molecular formula (elemental composition)] in the compound suggested that the lipopeptide was an open-ring structure and corresponded to the fragmentation pattern previously described by Eckelmann et al. (2018) (Figure 3). Overall, it is proposed that this compound group is comprised of four residues, including a peptide moiety of two serine residues (cyclic or open-ring) attached to two fatty acyl residues (varying in length and presence or absence of double bonds). From the described serratamolides it was assumed that the compounds described above contain L-serine residues and D-3-hydroyxy-fatty acyl residues.

**FIGURE 4:**
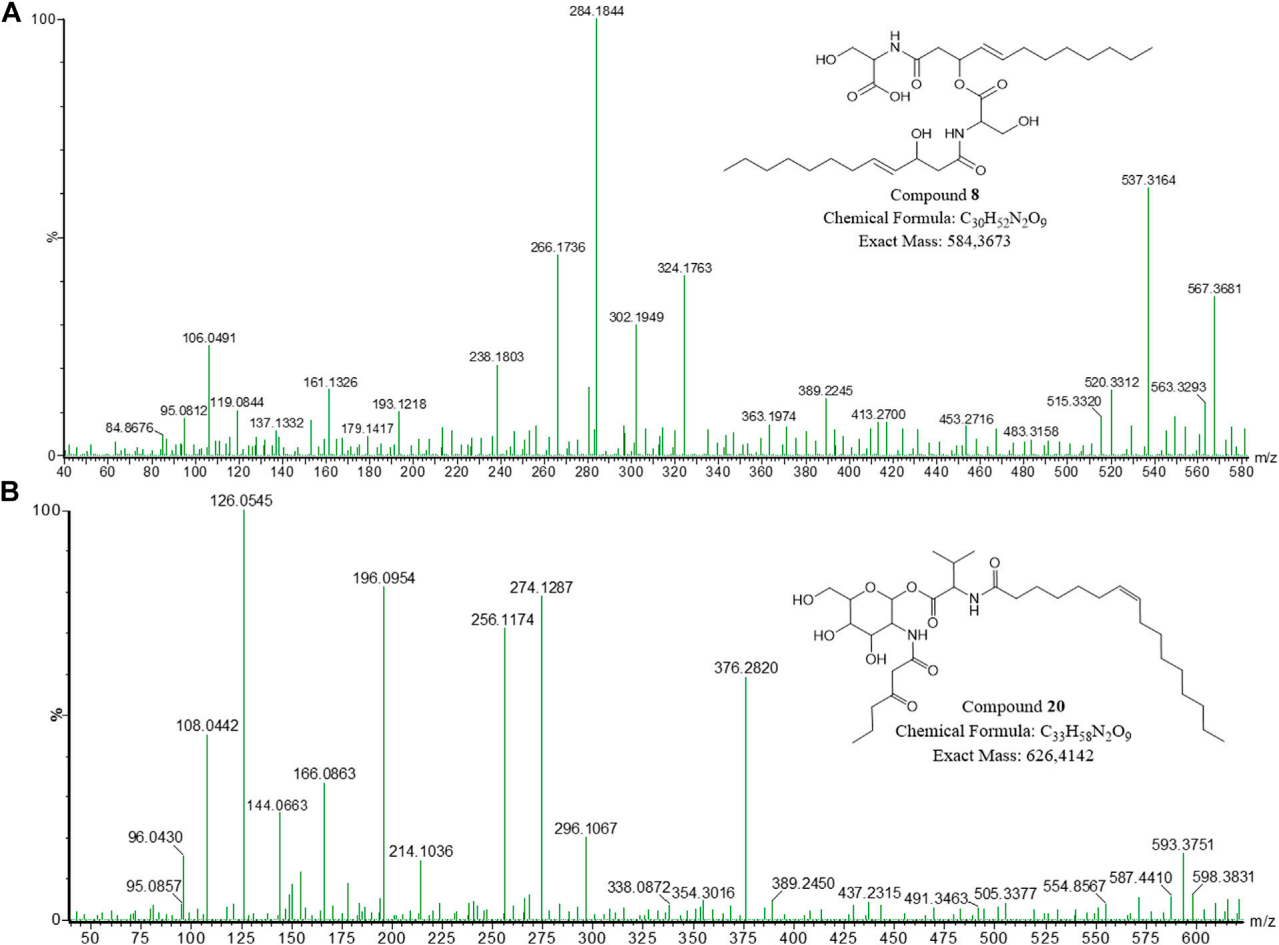
The fragmentation profiles and proposed structures of **(A)** open-ring serratamolide 585 (compound **8**) and **(B)** glucosamine derivative J (compound **20**).

The compound with *m/z* 585.374 [C_30_H_52_N_2_O_9_ + H]^+^ (compound **8**) did not cluster in the molecular network; however, it was detected at 12.46 min in the UPLC-MS^E^ data for the NP1 strain (Table 1; Figure 4A; Supplementary Figures S10, S22). The fragment with *m/z* 567.368 corresponded to a product of dehydration (removal of H, OH). A major fragment at *m/z* 284.185 was then observed, representing a dehydrated serine residue linked to an unsaturated hydroxydodecanoic acid (C_12:1_). In addition, the dehydration product of the *m/z* 284.185 fragment was detected at *m/z* 266.176. Similar to the previously described open-ring structures, the addition of a hydroxyl group [based on the experimental molecular formula (elemental composition)] in the compound suggested that the lipopeptide was an open-ring structure; however, this compound has not previously been reported in literature or databases (Norine and PubChem). The novel **8** homologue was thus proposed to comprised of two L-serine residues coupled to two unsaturated D-3-hydroxydodecanoic acyl groups (C_12:1_), correlating to described serratamolides (Figure 4A).

The F1 nodes with *m/z* 537.3147, 563.3290, 565.3455, 581.3405 and 609.3735 were found in the UPLC-MS^E^ data and corresponds to the sodiated adducts of the compounds **4**, **9**, **13**, **14**, **3**, and **11**, respectively, (Supplementary Table S1). In contrast, no structures were established for the F1 nodes with *m/z* 531.365 and 575.391.

#### Structural Elucidation of Glucosamine Derivatives

**FIGURE 5:**
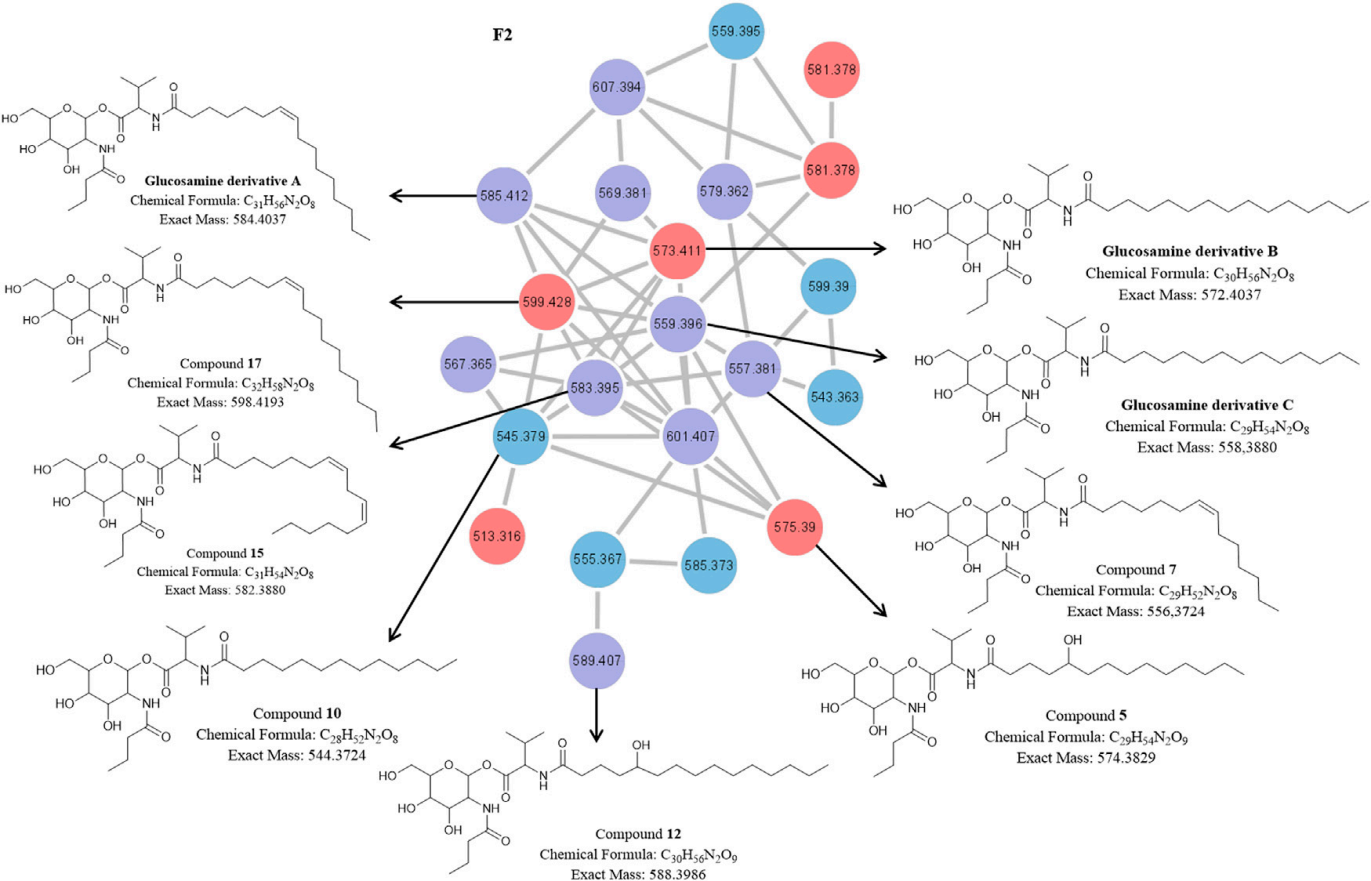
The molecular network of cluster F2 (magnified) with the corresponding structures of known and unknown glucosamine derivative homologues [previously identified by and adopted from Dwivedi et al. (2008)] detected in the P1 and NP1 crude extracts identified using UPLC-MS^E^ analysis. Red nodes = metabolites produced by the P1 strain; Blue nodes = metabolites produced by the NP1 strain; Purple nodes = metabolites produced by both P1 and NP1 strains.

The molecular network of the F2 cluster and corresponding structures for the known and novel compounds in the F2 cluster are presented in Figure 5. The F2 cluster (Figure 5) revealed nodes with *m/z* 585.4117 [C_31_H_56_N_2_O_8_ + H]^+^ (compound **16**) and 559.3953 [C_29_H_54_N_2_O_8_ + H]^+^ (compound **14**) corresponding to the peaks observed in the UPLC-MS^E^ data for the P1 and NP1 strains, while *m/z* 573.4136 [C_30_H_56_N_2_O_8_ + H]^+^ (compound **19**) corresponded to a peak observed in the UPLC-MS^E^ data for the P1 strain (Table 2; Supplementary Figures S11–S13, S23). The fragmentation profiles of these three compounds (**14**, **16**, and **19**) correspond to glucosamine derivative homologues that have previously been reported (Dwivedi et al., 2008). For all three compounds, the two major fragments were linked via an anomeric C-O bond between the hexose and valine amino acid residues. Therefore, **16** was proposed to be comprised of a hexose residue linked to butyric acyl group, which in turn was coupled to a valine residue linked to an unsaturated C_16:1_ fatty acid chain correlating to a previously described compound, glucosamine derivative A (Dwivedi et al., 2008) (Figure 5). The hexose was assumed to be glucose and the double bond within the fatty acyl chain of glucosamine derivative A was assumed to be a *cis* double bond corresponding to the structure previously described in literature (Dwivedi et al., 2008). Compound **19** was also proposed to be comprised of a glucose residue linked to a butyric acyl group, coupled to a valine residue linked to a saturated C_15_ fatty acid chain, thus correlating with the structure of glucosamine derivative B as described by Dwivedi et al. (2008) (Figure 5). Finally, **14** had a structure similar to the other two glucosamine derivatives. Compound **14** was also proposed to be comprised of a glucose residue linked to butyric acid, a valine residue linked to a saturated C_14_ fatty acid chain and thus the structure correlated to the glucosamine derivative C (Dwivedi et al., 2008) (Figure 5). Overall, it is proposed that this compound group is comprised of four residues, namely glucose, valine, butyric acid and a saturated or unsaturated β-fatty acid of varying length (C_14_ to C_16_).

Other F2 nodes (Figure 5) at *m/z* 557.3804 [C_29_H_52_N_2_O_8_ + H]^+^ (compound **7**), 583.3947 [C_31_H_54_N_2_O_8_ + H]^+^ (compound **15**) and 589.4056 [C_30_H_56_N_2_O_9_ + H]^+^ (compound **12**) had similar fragmentation profiles to **14**, **16**, and **19** and corresponded to peaks observed in the UPLC-MS^E^ data for P1 and NP1 strains (Table 2; Supplementary Figures S14–S16). In addition, F2 nodes at *m/z* 575.3902 [C_29_H_54_N_2_O_9_ + H]^+^ (compound **5**), 587.4268 [C_31_H_58_N_2_O_8_ + H]^+^ (compound **21**), and 599.4265 [C_32_H_58_N_2_O_8_ + H]^+^ (compound **17**) also had similar profiles to glucosamine derivative A to C and corresponded to peaks observed in the UPLC-MS^E^ data for the P1 strain, while another node at *m/z* 545.3787 [C_28_H_52_N_2_O_8_ + H]^+^ (compound **10**) corresponded to peaks observed in the UPLC-MS^E^ data for the NP1 strain (Table 2; Supplementary Figures S17–S20, S23). The cleavage of all seven compounds at the anomeric C-O bond resulted in two major fragments for each compound. Similar to the previously described known compounds **14**, **16**, and **19**, each compound had a major fragment of *m/z* 232.116 corresponding to the glucose/hexose residue linked to butyric acid, and dehydration products of the *m/z* 232.116 fragment were observed at *m/z* 214.107 and 196.097. Compounds **10** and **21** had a second major fragment of *m/z* 314.272 and 356.316, which corresponded to a valine residue linked to a saturated C_13_ and C_16_ fatty acyl chain, respectively. In addition, **7**, **15**, and **17** had a second major fragment of *m/z* 326.267, 352.282, and 368.316 which corresponded to a valine residue linked to an unsaturated C_14:1_, C_16:2_ and C_17:1_ fatty acid chain, respectively. Finally, **5** and **12** had a major fragment of *m/z* 344.280 and 358.296, which corresponded to a valine residue linked to a saturated C_14_ and C_15_ fatty acyl chain with an additional hydroxyl group in the fatty acyl residue. The presence of the hydroxyl group within the fatty acid moiety was further confirmed, as the loss of a hydroxyl group was observed within the fragmentation profile for both *m/z* 344.280 and 358.296 fragments, including *m/z* 326.269 and 340.286, respectively. In addition, the experimental molecular formula (elemental composition) predicted by the MassLynx 4.1 software package provided further indication of the addition of the hydroxyl group for both **5** and **12** (Table 2).

Typically, MS analysis is used in conjunction with other analytical methods, such as NMR, to determine the position of double bonds and hydroxyl groups within a structure (Eckelmann et al., 2018). However, the majority of the glucosamine derivative compounds were obtained in low quantities and could thus not be subjected to NMR analysis. A *cis* configuration was thus proposed for the double bonds in the fatty acid chain of the various unknown compounds, as the geometric isomerism of a double bond is typically a *cis* configuration in natural fatty acids (Kobelnik et al., 2018). Therefore, we propose that **7**, **15**, and **17** were novel glucosamine derivative homologues comprised of a glucose residue linked to butyric acyl, coupled to a valine residue linked to an unsaturated C_14:1_, C_16:2_, C_17:1_ fatty acyl chain, respectively (Figure 5). Compounds **10** and **21** were proposed to be comprised of a glucose residue linked to a butyric acyl, coupled to a valine residue linked to a saturated C_13_ and C_16_ fatty acyl chain, respectively (Figure 5). Finally, **5** and **12** were also proposed to be comprised of a glucose residue linked to butyric acyl group, coupled to a valine residue linked to a saturated C_14_ and C_15_ fatty acyl chain, respectively; however, the fatty acyl chain had an additional hydroxyl group (Figure 5). Overall, this compound group, correlating to the previous group, were proposed to be comprised of four residues, including glucose, valine, butyric acid and a fatty acid (Figure 5). Following an extensive literature and database search (Norine and PubChem), the identified structures (**5**, **7**, **10**, **12**, **15**, **17**, and **21**) and fragmentation patterns did not correspond to previously identified glucosamine derivatives (*N*-butylglucosamine ester derivatives) and are hypothesized to be novel glucosamine derivatives homologues. The F2 nodes with *m/z* 607.3924, 581.3781, 579.3623, and 567.3618 were found in the UPLC-MS^E^ data and corresponded to the sodiated adducts of compounds **16**, **14**, **7**, and **10**, respectively, (Supplementary Table S1). No structures were elucidated for the F2 nodes with *m/z* 513.316, 543.363, 555.367, 569.381, and 601.407.

In contrast, to the above mentioned glucosamine derivatives, an individual node (Figure 2) with *m/z* 627.4192 [C_33_H_58_N_2_O_9_ + H]^+^ (compound **20**) exhibited a unique fragmentation profile and corresponded to a peak at 16.28 min observed in the UPLC-MS^E^ data for the P1 strain (Table 2; Figure 4B; Supplementary Figures S21, S23). The fragmentation profile was unique as **20** did not have a major fragment of *m/z* 232.116 (corresponding to a hexose residue linked to butyric acyl group) that was common in all of the previously described glucosamine derivatives. Instead, a major fragment at *m/z* 274.129 was observed, which suggested a modification in the fragment corresponding to the hexose and butyric acyl moiety (Figure 4B). The major fragment at *m/z* 274.129 potentially corresponded to a hexose residue linked to an oxo-hexanoic acyl (rather than a butyric acyl), and a dehydration product of the *m/z* 274.129 fragment was observed at *m/z* 256.117. Furthermore, a second major fragment of *m/z* 354.302 was observed, which corresponded to an unsaturated C_16:1_ fatty acid chain. In comparison to the glucosamine derivative A (**16**) which also had an unsaturated C_16:1_ fatty acid chain, this glucosamine derivative had an additional C_2_H_3_O based on the experimental molecular formula (elemental composition) predicted using the MassLynx 4.1 software package. It is thus feasible that oxo-hexanoic acid was incorporated into the glucosamine derivative instead of butyric acid. In addition, **20** did not cluster with the glucosamine derivatives in the molecular network; rather, the compound formed an individual cluster and this further suggests the reduced relatedness of this compound to the glucosamine derivatives. Although NMR is required to confirm the structure, the putative novel congener of **20** was proposed to consist of glucose, oxo-hexanoic acid, valine and an unsaturated C_16:1_ fatty acid chain, which is distinctive from that of the previously described glucosamine derivatives (Figure 4B).

### Antimicrobial Activity

The bioactivity of the secondary metabolites produced by the P1 and NP1 strains was then investigated using disc diffusion, MIC and MBC assays. All the fractions collected during RP-HPLC analysis were subjected to antimicrobial testing using a disc diffusion assay against *E. faecalis* S1. It was found that *E. faecalis* S1 was susceptible (diameter range of 9.3 ± 2.1 to 19.3 ± 1.5 mm) to the fractions corresponding to the major compounds (higher molecular ion signal) **4**, **9**, **13**, **7**, **14**, and **16** (for both P1 and NP1), and the fraction of compound **2** collected from P1 crude extract (results not shown). Thereafter, the MIC and MBC assays were performed using five fractions (selected based on activity and quantity available), including fractions of the major compounds (higher molecular ion signal) **2**, **4**, **9**, **13**, and **16**. A summary of the MIC and MBC assay results are outlined in Table 3. It was observed that all three serratamolides (**4**, **9**, and **13**) (identified as outlined in *Structural Elucidation of Serratamolide Homologues*) exhibited identical activity against *E. faecalis* S1 with a MIC of 3 mg/ml and MBC of >3 mg/ml. In comparison, glucosamine derivative A (compound **16**; identified as outlined in *Structural Elucidation of Glucosamine Derivatives*) was found to have a lower MIC and MBC of 0.75 mg/ml and 3 mg/ml, respectively. Overall, however, the lowest MIC and MBC recorded against *E. faecalis* S1 was observed for prodigiosin (compound **2**; identified as outlined in *UPLC-MS* ^*E*^ *Analysis*), at <0.75 and 1.5 mg/ml, respectively, (Table 3).

**TABLE 3:** Minimum inhibition and bactericidal concentrations of selected fractions against the clinical *E. faecalis* S1 stain.

Compound no	Proposed identification (fatty acid chain length)	Purity	MIC (mg/ml)	MBC (mg/ml)
**4**	Serratamolide A (C_10_ + C_10_)	72%	3	>3
**9**	Serratamolide B (C_10_ + C_12:1_)	81%	3	>3
**13**	Serratamolide C (C_10_ + C_12_)	75%	3	>3
**16**	Glucosamine derivative A (C_16:1_)	80%	0.75	3
**2**	Prodigiosin	80%	<0.75	1.5

## Discussion

### Untargeted Metabolomics: UPLC-MS^E^ and Molecular Networking Analysis

This work reports on the identification of four molecular families of secondary metabolites associated with *Serratia* species, including serratiochelin A (**1**), prodigiosin (**2**), serratamolide homologues (**3**, **4**, **6**, **8**, **9**, **11**, **13**, and **18**) and glucosamine derivative congeners (**5**, **7**, **10**, **12**, **14**–**17**, and **19**–**21**) (refer to Figures 2–5). The structures of prodigiosin and serratiochelin A have previously been elucidated, where prodigiosin was described as a tripyrrole red pigment with an alkyl substituent (Figure 1A; Lee et al., 2011) and serratiochelin A was described as a low-molecular weight siderophore with high affinity for iron (Figure 1B; Khilyas et al., 2019). The structure of several of the serratamolide and glucosamine derivative metabolites detected in the current study has previously been elucidated, with serratamolides generally described as lipopeptides of two L-serine residues (cyclic or open-ring) linked to two fatty acid chains (Figure 1C) and glucosamine derivatives described as glucose amines consisting of glucose, valine, a fatty acid chain and butyric acid (Figure 1D; Dwivedi et al., 2008; Eckelmann et al., 2018). Moreover, based on their characteristic fragmentation patterns, as observed in the UPLC-MS^E^ data, eight serratamolides (including the putative structure of a novel open-ring serratamolide homologue) and 11 glucosamine derivatives (including the putative structures of eight novel glucosamine derivative congeners) were identified in this study and their structures were elucidated.

It is well-known that certain bacteria naturally produce various lipopeptide congeners which vary in fatty acid chain length, fatty acyl saturation or in the amino acyl residues of the peptide moiety (Das et al., 2008). This was similarly observed in the current study as the identified serratamolides, comprised of two L-serine residues linked to two fatty acyl residues, varied in the length (C_10_ to C_12_) and the presence or absence of double bonds (saturated or mono-unsaturated) in the fatty acyl chains and cyclization (open-ring or cyclic) of the peptide moiety. The glucosamine derivative congeners, comprised of glucose, valine, butyric acyl (or oxo-hexanoic acyl for **20**) and a fatty acyl residue, similarly varied in the length (C_13_ to C_17_), as well as the presence or absence of double bonds (saturated or unsaturated) or a hydroxyl group in the β-fatty acyl residue. During UPLC-MS^E^ analysis, the elution time (Rt) of the serratamolide and glucosamine derivative homologues was, as expected, dependent on the length of the β-hydroxy fatty acid chains of the compound. For instance, the serratamolides with shorter saturated fatty acid moieties, such as serratamolide A (C_10_ + C_10_) (**4**), eluted from the column before the serratamolides with longer saturated fatty acid moieties, such as serratamolide 571 (C_12_ + C_12_) (**18**) (refer to Table 1; Supplementary Figure S22). It was also observed that the Rt of the serratamolide and glucosamine derivative homologues was dependent on the saturation or unsaturation of the fatty acid moieties, for instance, as the unsaturated glucosamine derivative A (**16**) (C_16:1_) eluted before the corresponding saturated novel glucosamine derivative K (**21**) homologue (C_16_) with the same residues and fatty acid chain length (refer to Table 2; Supplementary Figure S23). Previous research has indicated that the presence and position of a double bond within the fatty acyl chain influences the Rt of a compound (Takashima et al., 2018) and the retention time of a mono-unsaturated fatty acid is reduced to that of the equivalent saturated fatty acid with two carbon units. A *cis* double bond causes a kink in the chain and reduces the actual molecular length by nearly two carbon units, thereby resulting in the elution of a C_18:1_ fatty acid just after a C_16_ fatty acid (Plattner et al., 1977; Takashima et al., 2018). It has also been reported that an open-chain lipopeptide is retained in the column for a shorter time period than the corresponding cyclic lipopeptide due to the increased polarity of the compound (due to the addition of the terminal COOH group) (Qiu et al., 2019), which clarifies for example, why the cyclic serratamolide C (C_10_ + C_12_) (**13**) eluted from the column after the open-chain serratamolide 561 (C_10_ + C_12_) (**6**) with the same fatty acid chain length. Furthermore, it was observed that the glucosamine derivatives with an additional hydroxyl group within the fatty acyl moieties were retained in the column for a shorter time period than the corresponding glucosamine derivative without a hydroxyl group, due to the increased polarity of the compound. For instance, the glucosamine derivative C (C_29_H_54_N_2_O_8_; **14**) with a C_14_ fatty acyl chain eluted from the column after the corresponding novel glucosamine derivative D homologue (C_29_H_55_N_2_O_9_; **5**) with a C_14_ fatty acyl chain that has an additional hydroxyl group. Overall, it was observed that the serratamolide and glucosamine derivative homologues separated as expected according to hydrophobicity (fatty acyl chain length) and chain saturation.

### Secondary Metabolic Profile of the *S. marcescens* P1 and NP1 Strains

Clements et al. (2019b) previously identified the secondary metabolites produced by the P1 and NP1 strains using UPLC-MS analysis, which proposed that the P1 strain produced prodigiosin and serratamolides (A, B, C, and E), while the NP1 strain produced glucosamine derivative A and serratamolides (A, B, C, and E). The use of RP-HPLC and UPLC-MS^E^ analysis in the current study revealed that the P1 strain produced four metabolic classes, including prodigiosin, serratiochelin A, serratamolide homologues (*n* = 6) and glucosamine derivative congeners (*n* = 10), while NP1 produced three metabolic classes, including serratiochelin A, serratamolide homologues (*n* = 8) and glucosamine derivative congeners (*n* = 6). It is hypothesized that the combination of RP-HPLC and UPLC-MS^E^ analysis was an effective approach to identify the minor metabolic constituents and elucidate the microbial metabolic profile of the environmental *Serratia* strains.

Genetically, secondary metabolites are synthesized by a number of gene clusters that can be identified using a bioinformatics approach (Romano et al., 2018). Certain gene clusters involved in secondary metabolism [such as non-ribosomal peptide synthetase (NRPS) gene clusters] may not be expressed under laboratory conditions and may be considered as “silent” gene clusters (Bode et al., 2002). Minor alterations in the cultivation conditions of a particular strain may, however, trigger the expression of the “silent” gene clusters and may allow for the discovery of novel small molecules (Bode et al., 2002). This phenomenon is known as the “one strain many compounds” (OSMAC) approach, which indicates that a single strain can produce various metabolites when grown under different media composition and cultivation conditions, leading to the activation of the “silent” metabolic pathways within the genome of the microbial strain (Bode et al., 2002; Romano et al., 2018). When comparing the culture conditions of Clements et al. (2019b) to the culture conditions employed in the current study, an increased production scale (from 100 ml to 1,500 ml) in baffled flasks, as well as the change in media components (from bactopeptone to peptone powder) was applied. Thus, while the combination of RP-HPLC and UPLC-MS^E^ analysis may have contributed to the detection of the minor metabolites; it is hypothesized that the altered culture conditions and media composition employed, may have activated “silent” NRPS clusters (such as the glucosamine derivatives in the P1 strain and the serratiochelin in both strains), thereby increasing the metabolic constituents produced by both the pigmented and non-pigmented *S. marcescens* strains.

Although genome mining of the P1 and NP1 strains was not within the scope of this study, Khilyas et al. (2019) sequenced the genome of a pigmented *S. marcescens* SM6 strain and used the antiSMASH 5.0 prediction tool to identify biosynthetic gene clusters involved in secondary metabolism within the strain. The authors identified five biosynthetic gene clusters involved in secondary metabolite production, including gene clusters responsible for the production of two siderophores (serratiochelin and chrysobactin), a thiopeptide, a serratamolide and a glucosamine derivative (Khilyas et al., 2019). It is possible that the P1 and NP1 strains possess similar NRPS gene clusters as both strains were able to produce serratiochelin, serratamolides and glucosamine derivatives. In addition, the bifurcated biosynthetic pathway responsible for the production of prodigiosin was additionally activated in the P1 strain (Lee et al., 2011). Following an extensive literature search, and to the best of the author’s knowledge, this is the first report of the co-production of all four secondary metabolic classes (including prodigiosin, serratiochelin A, serratamolides and glucosamine derivatives) by a *Serratia* strain.

### Antimicrobial Activity

Results from this study indicated that prodigiosin (**2**), serratamolide A (**4**), B (**9**), and C (**13**) homologues and glucosamine derivative A (**16**) and C (**14**) homologues, as well as the novel glucosamine derivative E (**7**) homologue exhibited activity against a clinical Gram-positive bacterial strain. The MIC and MBC assays further revealed that serratamolide A (**4**), B (**9**), and C (**13**) exhibited identical activity against *E. faecalis* S1, suggesting that the presence of a double bond or the increased length of one of the two fatty acid chains from C_10_ to C_12_ exhibited no additional antimicrobial potency against the Gram-positive strain. In comparison, glucosamine derivative A (**16**) was found to exhibit a lower MIC and MBC in comparison to the serratamolides. This indicates that *E. faecalis* S1 was more susceptible to the glucosamine derivative A (**16**) than the serratamolides. Overall, however, *E. faecalis* S1 was found to be the most susceptible to prodigiosin (**2**), as a lower MIC and MBC was obtained against the clinical strain in comparison to glucosamine derivative A (**16**) and the serratamolides (**4**, **9**, and **13**).

The potent activity of prodigiosin corresponds to previous research that similarly recorded activity of this pigmented compound against Gram-positive bacteria such as *Staphylococcus aureus* (*S. aureus*) and *Bacillus subtilis* (*B. subtilis*) (Darshan and Manonmani, 2015). It has also been suggested that prodigiosin is a hydrophobic stressor that disrupts the cell membrane and results in the leakage of intracellular products, such as K^+^ ions, amino acids, sugars and proteins (Suryawanshi et al., 2016). Furthermore, prodigiosin may interfere with cellular respiration and protein and ribosomal ribonucleic acid synthesis (Danevčič et al., 2016a; Danevcic et al., 2016b). Serratamolide A has similarly been reported to display activity toward Gram-positive bacteria, such as *S. aureus* strains (Kadouri and Shanks, 2013). It has additionally been reported that serratamolide A (serrawettin W1) increased the rate of movement of K^+^ and H^+^ across the membrane of *S. aureus* at a concentration of 10 μg/ml, while no leakage of “260 nm-absorbing products” (such as nucleic acids), membrane permeability or cell growth inhibition was observed at this concentration (Deol et al., 1973). Currently, one study has reported on the antimicrobial activity of glucosamine derivative homologues (A to C) toward *Mycobacterium diernhoferi*; however, little is known about the mechanism of action of these metabolites (Dwivedi et al., 2008). Moreover, Hage-Hülsmann et al. (2018) described the enhanced antimicrobial activity of serrawettin W1 (serratamolide A) and prodigiosin in combination, in comparison to the individual bioactive metabolites, against *Corynebacterium glutamicum*. The authors hypothesized that the co-production of serrawettin W1 and prodigiosin may provide a competitive advantage to the producing strain due to the increased bioactive potency, while also aiding in the colonization and biofilm development of the producing strain by inhibiting or delaying the growth of competing microorganisms. Thus, while extensive research is required to investigate the ecological role of co-producing prodigiosin, serratamolides and glucosamine derivatives; the combined production of these bioactive metabolites by *S. marcescens* may provide a competitive advantage to the producing strain (Darshan and Manonmani, 2015; Hage-Hülsmann et al., 2018). Moreover, as indicated in Clements et al. (2019b), the metabolites in the P1 strain may exhibit a higher antimicrobial effect in comparison to the metabolites in the NP1 strain, as the secondary metabolites in the P1 crude extract were effective against 81% of the test microbial organisms, in comparison to the 67% antimicrobial efficacy recorded for the NP1 strain.

## Conclusion

The use of RP-HPLC for chromatographic fractionation, high resolution ESI-MS, UPLC-MS^E^ and molecular networking analysis allowed for the identification and elucidation of 21 secondary metabolites (combined total for P1 and NP1 strains), including serratamolide homologues, glucosamine derivative homologues, prodigiosin (P1) and serratiochelin A. Moreover, the putative structure of an open-ring serratamolide homologue and putative structures of eight novel glucosamine derivative congeners were elucidated. The structural relatedness of the novel open-ring serratamolide and glucosamine derivative congeners to the known serratamolides and glucosamine derivatives, respectively, was revealed using molecular networking and fragmentation patterns of the UPLC-MS^E^ data. This approach thus proved vital for metabolite identification and characterization. The identified metabolic families exhibited structural diversity and well-ordered chromatographic behaviors due to structural modifications of fatty acid chain lengths, open-ring or cyclic structures and the degree of saturation (saturated or mono-unsaturated) of the fatty acid chain. Moreover, seven fractions collected during RP-HPLC, with major molecular ions corresponding to prodigiosin (**2**), serratamolide A (**4**), B (**9**), and C (**13**) homologues and glucosamine derivative A (**16**), C (**14**), and E (**7**) homologues, displayed antimicrobial activity against the clinical *E. faecalis* S1 strain using the disc diffusion assay. However, the MIC and MBC assays revealed that the three serratamolides [A (**4**), B (**9**), and C (**13**)] exhibited the lowest antimicrobial potency (same MIC and MBC values for all three) of the metabolites tested, and highlighted that presence of a double bond or the change in length of a fatty acid chain (C_10_ to C_12_) of the serratamolides provided no additional antimicrobial potency against the Gram-positive strain. Prodigiosin (**2**) however, exhibited the greatest antimicrobial potency, followed by glucosamine derivative A (**16**). This study thus highlights the importance of *Serratia* species as a source of novel antimicrobial compounds for potential therapeutic application. It is recommended that future research investigate the mode of action and synergism of the bioactive secondary metabolites produced by these two *S. marcescens* strains. In addition, the use of whole genome sequencing of the P1 and NP1 strains and subsequent genome mining of NRPS gene clusters is a promising future endeavour to identify additional secondary metabolites not actively being produced under typical cultivation conditions.

## Data Availability

The original contributions presented in the study are included in the article/Supplementary Material, further inquiries can be directed to the corresponding author.

## References

[B1] Bode H. B. Bethe B. Höfs R. Zeeck A. (2002). Big effects from small changes: possible ways to explore nature’s chemical diversity. ChemBioChem 3 (7), 619–627. 10.1002/1439-7633(20020703)3:7<619::AID-CBIC619>3.0.CO;2-9 12324995

[B2] Clements T. Ndlovu T. Khan S. Khan W. (2019a). Biosurfactants produced by *Serratia* species: classification, biosynthesis, production and application. Appl. Microbiol. Biotechnol. 103, 589–602. 10.1007/s00253-018-9520-5 30456577

[B3] Clements T. Ndlovu T. Khan W. (2019b). Broad-spectrum antimicrobial activity of secondary metabolites produced by *Serratia marcescens* strains. Microbiol. Res. 229, 126329. 10.1016/j.micres.2019.126329 31518853

[B4] Danevčič T. Vezjak M. B. Tabor M. Zorec M. Stopar D. (2016b). Prodigiosin induces autolysins in actively grown *Bacillus subtilis* cells. Front. Microbiol. 7, 27. 10.3389/fmicb.2016.00027 26858704 PMC4729933

[B5] Danevčič T. Vezjak M. B. Zorec M. Stopar D. (2016a). Prodigiosin–a multifaceted *Escherichia coli* antimicrobial agent. PLoS One 11 (9), 1–13. 10.1371/journal.pone.0162412 PMC5017725 27612193

[B6] Darshan N. Manonmani H. K. (2015). Prodigiosin and its potential applications. J. Food Sci. Technol. 52, 5393–5407. 10.1007/s13197-015-1740-4 26344956 PMC4554646

[B7] Das P. Mukherjee S. Sen R. (2008). Antimicrobial potential of a lipopeptide biosurfactant derived from a marine *Bacillus circulans* . J. Appl. Microbiol. 104 (6), 1675–1684. 10.1111/j.1365-2672.2007.03701.x 18194244

[B8] Deol B. S. Bermingham M. A. Still J. L. Haydon D. A. Gale E. F. (1973). The action of serratamolide on ion movement in lipid bilayers and biomembranes. Biochim. Biophys. Acta Biomembr. 330 (2), 192–195. 10.1016/0005-2736(73)90224-1 4591125

[B9] Dwivedi D. Jansen R. Molinari G. Nimtz M. Johri B. N. Wray V. (2008). Antimycobacterial serratamolides and diacyl peptoglucosamine derivatives from *Serratia* sp. J. Nat. Prod. 71 (4), 637–641. 10.1021/np7007126 18303848

[B10] Eckelmann D. Spiteller M. Kusari S. (2018). Spatial-temporal profiling of prodiginines and serratamolides produced by endophytic *Serratia marcescens* harbored in *Maytenus serrata* . Sci. Rep. 8, 1–15. 10.1038/s41598-018-23538-5 29588473 PMC5869619

[B11] European Committee on Antimicrobial Susceptibility Testing. (2018). Breakpoint tables for interpretation of MICs and zone diameters. Available at: http://www.eucast.org/fileadmin/src/media/PDFs/EUCAST_files/Breakpoint_tables/v_8.0_Breakpoint_Tables.pdf (Accessed July 10, 2020).

[B12] Fu Y. Luo J. Qin J. Yang M. (2019). Screening techniques for the identification of bioactive compounds in natural products. J. Pharm. Biomed. Anal. 168, 189–200. 10.1016/j.jpba.2019.02.027 30825802

[B13] Grimont F. Grimont P. A. D. (2015). “Serratia,” in Bergey’s manual of systematics of archaea and bacteria. Editors DeVos P. Chun J. Dedysh S. Hedlund B. Kämpfer P. Raine F. (Hoboken, NJ: John Wiley & Sons), 1–81.

[B14] Hage-Hülsmann J. Grünberger A. Thies S. Santiago-Schübel B. Klein A. S. Pietruszka J. (2018). Natural biocide cocktails: combinatorial antibiotic effects of prodigiosin and biosurfactants. PLoS One 13 (7), e0200940. 10.1371/journal.pone.0200940 30024935 PMC6053208

[B15] Kadouri D. E. Shanks R. M. (2013). Identification of a methicillin-resistant *Staphylococcus aureus* inhibitory compound isolated from *Serratia marcescens* . Res. Microbiol. 164 (8), 821–826. 10.1016/j.resmic.2013.06.002 23791620 PMC3770767

[B16] Khilyas I. V. Tursunov K. A. Shirshikova T. V. Kamaletdinova L. K. Matrosova L. E. Desai P. T. (2019). Genome sequence of pigmented siderophore-producing strain *Serratia marcescens* SM6. Microbiol. Resour. Announc. 8 (18), e00247–19. 10.1128/MRA.00247-19 31048396 PMC6498229

[B17] Knolhoff A. M. Zheng J. McFarland M. A. Luo Y. Callahan J. H. Brown E. W. (2015). Identification and structural characterization of naturally-occurring broad-spectrum cyclic antibiotics isolated from *Paenibacillus* . J. Am. Soc. Mass Spectr. 26 (10), 1768–1779. 10.1007/s13361-015-1190-2 26250559

[B18] Kobelnik M. Fontanari G. G. Ribeiro C. A. Crespi M. S. (2018). Evaluation of thermal behavior and chromatographic characterization of oil extracted from seed of *Pittosporum undulatum* . J. Therm. Anal. Calorim. 131 (1), 371–378. 10.1007/s10973-017-6763-9

[B19] Kuo Y. H. Liang T. W. Liu K. C. Hsu Y. W. Hsu H. C. Wang S. L. (2011). Isolation and identification of a novel antioxidant with antitumour activity from *Serratia ureilytica* using squid pen as fermentation substrate. Mar. Biotechnol. 13 (3), 451–461. 10.1007/s10126-010-9316-9 20922553

[B20] Lee J. S. Kim Y. S. Park S. Kim J. Kang S. J. Lee M. H. (2011). Exceptional production of both prodigiosin and cycloprodigiosin as major metabolic constituents by a novel marine bacterium, *Zooshikella rubidus* S1-1. Appl. Environ. Microbiol. 77 (14), 4967–4973. 10.1128/AEM.01986-10 21642414 PMC3147376

[B21] Magalhães L. Nitschke M. (2013). Antimicrobial activity of rhamnolipids against *Listeria monocytogenes* and their synergistic interaction with nisin. Food Control 29 (1), 138–142. 10.1016/j.foodcont.2012.06.009

[B22] Matsuyama T. Fujita M. Yano I. (1985). Wetting agent produced by *Serratia marcescens* . FEMS Microbiol. Lett. 28 (1), 125–129. 10.1111/j.1574-6968.1985.tb00777.x

[B23] Matsuyama T. Kaneda K. Nakagawa Y. Isa K. Hara-Hotta H. Yano I. (1992). A novel extracellular cyclic lipopeptide which promotes flagellum-dependent and -independent spreading growth of *Serratia marcescens* . J. Bacteriol. 174 (6), 1769–1776. 10.1128/jb.174.6.1769-1776.1992 1548227 PMC205777

[B24] Matsuyama T. Murakami T. Fujita M. Fujita S. Yano I. (1986). Extracellular vesicle formation and biosurfactant production by *Serratia marcescens* . Microbiol 132 (4), 865–875. 10.1099/00221287-132-4-865

[B25] Motley J. L. Stamps B. W. Mitchell C. A. Thompson A. T. Cross J. You J. (2016). Opportunistic sampling of roadkill as an entry point to accessing natural products assembled by bacteria associated with non-anthropoidal mammalian microbiomes. J. Nat. Prod. 80, 598–608. 10.1021/acs.jnatprod.6b00772 28335605 PMC5368682

[B26] Ndlovu T. Rautenbach M. Vosloo J. A. Khan S. Khan W. (2017). Characterisation and antimicrobial activity of biosurfactant extracts produced by *Bacillus amyloliquefaciens* and *Pseudomonas aeruginosa* isolated from a wastewater treatment plant. AMB Express 7 (108), 1–19. 10.1186/s13568-017-0363-8 28571306 PMC5451374

[B27] Philippus A. C. Zatelli G. A. Wanke T. Barros M. G. D. A. Kami S. A. Lhullier C. (2018). Molecular networking prospection and characterization of terpenoids and C 15-acetogenins in Brazilian seaweed extracts. RSC Adv. 8 (52), 29654–29661. 10.1039/C8RA02802H PMC9085288 35547298

[B28] Plattner R. D. Spencer G. F. Kleiman R. (1977). Triglyceride separation by reverse phase high performance liquid chromatography. J. Am. Oil Chem. Soc. 54 (11), 511–515. 10.1007/BF02909070

[B29] Qiu S. Avula B. Guan S. Ravu R. R. Wang M. Zhao J. (2019). Identification of fusaricidins from the antifungal microbial strain *Paenibacillus* sp. MS2379 using ultra-high performance liquid chromatography coupled to quadrupole time-of-flight mass spectrometry. J. Chromatogr. A. 1586, 91–100. 10.1016/j.chroma.2018.12.007 30558848

[B30] Romano S. Jackson S. A. Patry S. Dobson A. D. (2018). Extending the “one strain many compounds” (OSMAC) principle to marine microorganisms. Mar. Drugs 16 (7), 244. 10.3390/md16070244 PMC6070831 30041461

[B31] Seyedsayamdost M. R. Cleto S. Carr G. Vlamakis H. João Vieira M. Kolter R. (2012). Mixing and matching siderophore clusters: structure and biosynthesis of serratiochelins from *Serratia* sp. V4. J. Am. Chem. Soc. 134 (33), 13550–13553. 10.1021/ja304941d 22830960 PMC3424848

[B32] Soenens A. Imperial J. (2019). Biocontrol capabilities of the genus *Serratia* . Phytochem. Rev. 2019, 1–11. 10.1007/s11101-019-09657-5

[B33] Stankovic N. Senerovic L. Ilic-Tomic T. Vasiljevic B. Nikodinovic-Runic J. (2014). Properties and applications of undecylprodigiosin and other bacterial prodigiosins. Appl. Microbiol. Biotechnol. 98, 3841–3858. 10.1007/s00253-014-5590-1 24562326

[B34] Su C. Xiang Z. Liu Y. Zhao X. Sun Y. Li Z. (2016). Analysis of the genomic sequences and metabolites of *Serratia surfactantfaciens* sp. nov. YD25^T^ that simultaneously produces prodigiosin and serrawettin W2. BMC Genom 17, 865. 10.1186/s12864-016-3171-7 PMC5094094 27809759

[B35] Suryawanshi R. K. Patil C. D. Koli S. H. Hallsworth J. E. Patil S. V. (2016). Antimicrobial activity of prodigiosin is attributed to plasma membrane damage. Nat. Prod. Res. 31 (5), 572–577. 10.1080/14786419.2016.1195380 27353356

[B36] Takashima S. Toyoshi K. Shimozawa N. (2018). Analyses of the fatty acid separation principle using liquid chromatography-mass spectrometry. Med. Mass Spectrom. 2, 1–13. 10.24508/mms.2018.06.002

[B37] Thies S. Santiago-Schübel B. Kovačić F. Rosenau F. Hausmann R. Jaeger K. E. (2014). Heterologous production of the lipopeptide biosurfactant serrawettin W1 in *Escherichia coli* . J. Biotechnol. 181, 27–30. 10.1016/j.jbiotec.2014.03.037 24732103

[B38] Tsugawa H. Cajka T. Kind T. Ma Y. Higgins B. Ikeda K. (2015). MS-DIAL: data-independent MS/MS deconvolution for comprehensive metabolome analysis. Nat. Methods 12 (6), 523–526. 10.1038/nmeth.3393 25938372 PMC4449330

[B39] Wang M. Carver J. J. Phelan V. V. Sanchez L. M. Garg N. Peng Y. (2016). Sharing and community curation of mass spectrometry data with global natural products social molecular networking. Nat. Biotechnol. 34 (8), 828–837. 10.1038/nbt.3597 27504778 PMC5321674

[B40] Wasserman H. H. Keggi J. J. McKeon J. E. (1961). Serratamolide, a metabolic product of *Serratia* . J. Am. Chem. Soc. 83 (19), 4107–4108. 10.1021/ja01480a046

[B41] Williamson N. R. Fineran P. C. Leeper F. J. Salmond G. P. (2006). The biosynthesis and regulation of bacterial prodiginines. Nat. Rev. Microbiol. 4 (12), 887–899. 10.1038/nrmicro1531 17109029

[B42] Yasir M. Dutta D. Willcox M. D. (2019). Comparative mode of action of the antimicrobial peptide melimine and its derivative Mel4 against *Pseudomonas aeruginosa* . Sci. Rep. 9 (1), 1–12. 10.1038/s41598-019-42440-2 31068610 PMC6506473

[B43] Yip C. H. Yarkoni O. Ajioka J. Wan K. L. Nathan S. (2019). Recent advancements in high-level synthesis of the promising clinical drug, prodigiosin. Appl. Microbiol. Biotechnol. 103 (4), 1667–1680. 10.1007/s00253-018-09611-z 30637495

