# Supplementary material for: A Metabolomics and Molecular Networking Approach to Elucidate the Structures of Secondary Metabolites Produced by Serratia marcescens Strains
Source: Front Chem. 2021 Mar 16;9:633870. doi: 10.3389/fchem.2021.633870 (PMC8007976; doi:10.3389/fchem.2021.633870)
Supplement: Supplementary file 1 [file table1.docx]

**Contribution to the Field Statement**

**A metabolomics and molecular networking approach to elucidate the structures of the secondary metabolites produced by *Serratia marcescens* strains**

Tanya Clements^1^, Marina Rautenbach^2^, Thando Ndlovu^1^, Sehaam Khan^3^ and Wesaal Khan^1^*

^1^Department of Microbiology, Faculty of Science, Stellenbosch University, Private Bag X1, Stellenbosch, 7602, South Africa

^2^Department of Biochemistry, Faculty of Science, Stellenbosch University, Private Bag X1, Stellenbosch, 7602, South Africa

^3^Faculty of Health Sciences, University of Johannesburg, PO Box 17011, Doornfontein, 2028, South Africa

**Contribution to the Field Statement (200 words)**

The combined use of untargeted ultra-performance liquid chromatography coupled to tandem mass spectrometry (UPLC-MS^e^) and molecular networking was a high-throughput method of providing crucial insight into the secondary metabolic profiles of pigmented and non-pigmented *Serratia* species. Furthermore, the use of chromatographic fractionation and UPLC-MS^e^ analysis proved to be a powerful tool for the detection of minor secondary metabolic constituents produced by *Serratia* strains. The integrated approach applied in this study thus provided new knowledge on the chemical structures of novel serratamolide and glucosamine derivative congeners and is the first report of the co-production of prodigiosin, serratiochelin A, serratamolides and glucosamine derivatives by a *Serratia* strain. A broth microdilution assay revealed that serratamolide A, B and C exhibited identical activity against a clinical *Enterococcus faecalis* (*E. faecalis*) strain, indicating that the variations in the fatty acid moiety (i.e lengths of C_10_ to C_12_ and the presence of a double bond in one of the two fatty acid chains) of the serratamolides exhibited no additional antimicrobial potency against the Gram-positive bacterium. Moreover, glucosamine derivative A and prodigiosin were found to be more potent against *E. faecalis* compared to the serratamolides, suggesting that these compounds are promising antimicrobial candidates for future drug development and therapeutic application.
