# Supplementary material for: A Metabolomics and Molecular Networking Approach to Elucidate the Structures of Secondary Metabolites Produced by Serratia marcescens Strains
Source: Front Chem. 2021 Mar 16;9:633870. doi: 10.3389/fchem.2021.633870 (PMC8007976; doi:10.3389/fchem.2021.633870)
Supplement: Supplementary file 2 [file table2.docx]

**Supplementary data**

**A metabolomics and molecular networking approach to elucidate the structures of the secondary metabolites produced by *Serratia marcescens* strains**

Tanya Clements^1^, Marina Rautenbach^2^, Thando Ndlovu^1^, Sehaam Khan^3^ and Wesaal Khan^1^*

^1^Department of Microbiology, Faculty of Science, Stellenbosch University, Private Bag X1, Stellenbosch, 7602, South Africa

^2^BioPep^TM^ Peptide group, Department of Biochemistry, Faculty of Science, Stellenbosch University, Private Bag X1, Stellenbosch, 7602, South Africa

^3^Faculty of Health Sciences, University of Johannesburg, PO Box 17011, Doornfontein, 2028, South Africa

**Running title:** Metabolites produced by *Serratia marcescens*

***Correspondence**: Wesaal Khan; Phone: +27 21 808 5804; E-mail: [wesaal@sun.ac.za](mailto:wesaal@sun.ac.za)

**Table S1**. Summary of the compounds in the P1 and NP1 crude extracts that were identified using RP-HPLC and ESI-MS analysis.

| **Compound no.** | **Crude extract** | **RP-HPLC**  **Rt (min)** | **Proposed compound**  **identity (literature)** | ***m/z* [M+H]^+^** | ***m/z* [M+Na]^+^** | ***m/z* [M+K]^+^** | **Reference** |
| --- | --- | --- | --- | --- | --- | --- | --- |
| **1** | P1, NP1 | 8.8, 8.7 | Serratiochelin A/ Serranticin | 430.1609 | 452.1430 | 468.1061 | Seyedsayamdost et al. 2012 |
| **2** | P1 | 20.9 | Prodigiosin | 324.2073 | N/D | N/D | Lee et al. 2011 |
| **3** | P1 | 20.9 | Open-ring serratamolide B | 559.3600 | 581.3405 | 597.3135 | Eckelmann et al. 2018 |
| **4** | P1, NP1 | 22.0 | Serrawettin W1/ serratamolide A | 515.3331 | 537.3147 | 553.2894 | Dwivedi et al. 2008 |
| **5** | P1 | 22.0 | Unidentified | 575.3902 | 597.3736 | 613.3528 | N/D |
| **6** | P1, NP1 | 22.0 | Open-ring serratamolide C | 561.3749 | 583.3563 | 599.3333 | Eckelmann et al. 2018 |
| **7** | P1, NP1 | 24.1 | Unidentified | 557.3804 | 579.3623 | 595.3395 | N/D |
| **8** | NP1 | 24.6 | Unidentified | 585.3738 | 607.3548 | 623.3301 | N/D |
| **9** | P1, NP1 | 24.5, 24.6 | Serratamolide B | 541.3485 | 563.3290 | 579.3591 | Dwivedi et al. 2008 |
| **10** | NP1 | 24.6 | Unidentified | 545.3787 | 567.3618 | 583.3265 | N/D |
| **11** | NP1 | 24.6 | Open-ring serratamolide 587 | 587.3908 | 609.3735 | 625.3573 | Eckelmann et al. 2018 |
| **12** | P1, NP1 | 26.4, 26.5 | Unidentified | 589.4056 | 611.3892 | 627.3608 | N/D |
| **13** | P1, NP1 | 26.4, 26.5 | Serratamolide C | 543.3644 | 565.3455 | 581.3188 | Dwivedi et al. 2008 |
| **14** | P1, NP1 | 28.8 | Glucosamine derivative C | 559.3953 | 581.3781 | 597.3525 | Dwivedi et al. 2008 |
| **15** | P1, NP1 | 29.7 | Unidentified | 583.3947 | 605.3761 | 621.3491 | N/D |
| **16** | P1, NP1 | 29.8, 29.7 | Glucosamine derivative A | 585.4117 | 607.3924 | 623.3680 | Dwivedi et al. 2008 |
| **17** | P1 | 31.1 | Unidentified | 599.4265 | 621.4111 | 637.3778 | N/D |
| **18** | P1 | 31.8 | Serratamolide 571 | 571.3931 | 593.3783 | 609.4060 | Eckelmann et al. 2018 |
| **19** | P1 | 31.8 | Glucosamine derivative B | 573.4136 | 595.3904 | 611.3514 | Dwivedi et al. 2008 |
| **20** | P1 | 31.8 | Unidentified | 627.4192 | 649.4021 | 665.3962 | N/D |
| **21** | P1 | 34.3, 34.5 | Unidentified | 587.4268 | 609.4103 | 625.3834 | N/D |

N/D – not detected

**Figure S1**. Fragmentation profile and structure of the compound ion [M+H]^+^ at *m/z* 430.1609.

**Table S2**. Summary of the theoretical and experimental fragmentation ions and mass error for the compound ion [M+H]^+^ at *m/z* 430.1602.

| **Proposed ion** | | **Theoretical**  **singly charged  *m/z*** | **Experimental  singly charged  *m/z*** | **Mass error  (ppm)** |
| --- | --- | --- | --- | --- |
|  | [M + H] | 430.1614 | 430.1609 | 1.5 |
| **1** | [M – C_7_H_6_O_3_] | 294.1454 | 294.1455 | -0.3 |
| **2** | [M – C_7_H_7_NO_3_] | 277.1188 | 277.1194 | -2.2 |
| **3** | [M – C_10_H_14_N_2_O_3_] | 220.0610 | 220.0607 | 1.4 |
| **4** | [M – C_11_H_9_NO_4_] | 211.1082 | 211.1077 | 2.4 |
| **5** | [M – C_13_H_25_NO_5_] | 194.0817 | 194.0804 | 6.7 |
| **6** | [M – C_11_H_12_N_2_O_4_] | 192.0660 | 192.0650 | 5.2 |
| **7** | [M – C_14_H_19_N_3_O_4_] | 137.0239 | 137.0234 | 3.7 |

**Figure S2**. Fragmentation profile and structure of the compound ion [M+H]^+^ at *m/z* 324.2073.

| **Proposed ion** | | **Theoretical**  **singly charged  *m/z*** | **Experimental  singly charged  *m/z*** | **Mass error  (ppm)** |
| --- | --- | --- | --- | --- |
|  | [M + H] | 324.2076 | 324.2073 | 1.4 |
| **1** | [M – CH_4_] | 309.1841 | 309.1836 | 1.6 |
| **2** | [M – CH_4_O] | 292.1813 | 292.1797 | 5.5 |
| **3** | [M – C_5_H_12_] | 252.1137 | 252.1140 | -1.2 |
| **4** | [M – C_4_H_10_] | 266.1293 | 266.1296 | -1.1 |

**Table S3**. Summary of the theoretical and experimental fragmentation ions and mass error for the compound ion [M+H]^+^ at *m/z* 324.2073.

**Figure S3**. Fragmentation profile and structure of the compound ion [M+H]^+^ at *m/z* 515.3331.

**Table S4**. Summary of the theoretical and experimental fragmentation ions and mass error for the compound ion [M+H]^+^ at *m/z* 515.3331.

| **Proposed ion** | | **Theoretical**  **singly charged  *m/z*** | **Experimental  singly charged  *m/z*** | **Mass error  (ppm)** |
| --- | --- | --- | --- | --- |
|  | [M + H] | 515.3332 | 515.3331 | 0.5 |
| **1** | [M – H, OH] | 497.3227 | 497.3239 | -2.4 |
| **2** | [M – C=O] | 487.3383 | 487.3371 | 2.5 |
| **3** | [M – C=O – H, OH] | 469.3277 | 469.3275 | 0.4 |
| **4** | [**3** – H, OH] | 451.3172 | 451.3146 | 5.8 |
| **5** | [M – Ser – H, OH] | 410.2906 | 410.2909 | -0.7 |
| **6** | [M – C_13_H_25_NO_5_] | 276.1811 | 276.1823 | -4.3 |
| **7** | [**6** – H, OH] | 258.1705 | 258.1679 | 10.1 |
| **8** | [**7** – H, OH] | 240.1600 | 240.1595 | 2.1 |
| **9** | [**8** – C=O] | 212.1650 | 212.1662 | -5.7 |
| **10** | [**8** – Ser] | 153.1279 | 153.1276 | 2.0 |
| **11** | [**6** – C_10_H_18_O_2_] | 106.0504 | 106.0499 | 4.7 |

**Figure S4**. Fragmentation profile and structure of the compound ion [M+H]^+^ at *m/z* 541.3485.

**Table S5**. Summary of the theoretical and experimental fragmentation ions and mass error for the compound ion [M+H]^+^ at *m/z* 541.3485.

| **Proposed ion** | | **Theoretical**  **singly charged  *m/z*** | **Experimental  singly charged  *m/z*** | **Mass error  (ppm)** |
| --- | --- | --- | --- | --- |
|  | [M + H] | 541.3488 | 541.3485 | 0.6 |
| **1** | [M – H, OH] | 523.3384 | 523.3371 | 2.5 |
| **2** | [M – C=O] | 513.3540 | 513.3548 | -1. 6 |
| **3** | [M – Ser – H, OH] | 436.3063 | 436.3065 | -0.5 |
| **4** | [M – C_13_H_25_NO_5_] | 302.1967 | 302.1981 | -4.6 |
| **5** | [**4** – H, OH] | 284.1862 | 284.1857 | 1.8 |
| **6** | [**5** – H, OH] | 266.1756 | 266.1749 | 2.6 |
| **7** | [M – C_15_H_27_NO_3_] | 276.1811 | 276.1795 | 5.8 |
| **8** | [**7** – H, OH] | 258.1705 | 258.1703 | 0.8 |
| **9** | [**8** – H, OH] | 240.1599 | 240.1597 | 0.8 |
| **10** | [**9** – C=O] | 212.1650 | 212.1630 | 9.4 |
| **11** | [**9** – Ser] | 153.1279 | 153.1270 | 5.9 |
| **12** | [**7** – C_10_H_18_O_2_] | 106.0504 | 106.0502 | 1. 9 |
| **13** | [**6** – Ser] | 179.1436 | 179.1452 | -8.9 |

**Figure S5**. Fragmentation profile and structure of the compound ion [M+H]^+^ at *m/z* 543.3644.

**Table S6**. Summary of the theoretical and experimental fragmentation ions and mass error for the compound ion [M+H]^+^ at *m/z* 543.3644.

| **Proposed ion** | | **Theoretical**  **singly charged  *m/z*** | **Experimental  singly charged  *m/z*** | **Mass error  (ppm)** |
| --- | --- | --- | --- | --- |
|  | [M + H] | 543.3645 | 543.3644 | 0.2 |
| **1** | [M – H, OH] | 525.3540 | 525.3543 | -0.6 |
| **2** | [M – C=O] | 515.3696 | 515.3705 | -1.8 |
| **3** | [M – C_13_H_25_NO_5_] | 304.2124 | 304.2116 | 2.6 |
| **4** | [**3** – H, OH] | 286.2018 | 286.2005 | 4.5 |
| **5** | [**4** – H, OH] | 268.1912 | 268.1912 | 0.0 |
| **6** | [M – C_15_H_29_NO_3_] | 276.1811 | 276.1811 | 0.0 |
| **7** | [**6** – H, OH] | 258.1705 | 258.1706 | -0.4 |
| **8** | [**7** – H, OH] | 240.1599 | 240.1593 | 2.5 |
| **9** | [**8** – C=O] | 212.1650 | 212.1637 | 6.1 |
| **10** | [**8** – Ser] | 153.1279 | 153.1291 | -7.8 |
| **11** | [**6** – C_10_H_18_O_2_] | 106.0504 | 106.0492 | 11.3 |
| **12** | [**5** – Ser] | 181.1592 | 181.1591 | 0.6 |

**Figure S6**. Fragmentation profile and structure of the compound ion [M+H]^+^ at *m/z* 561.3749.

**Table S7**. Summary of the theoretical and experimental fragmentation ions and mass error for the compound ion [M+H]^+^ at *m/z* 561.3749.

| **Proposed ion** | | **Theoretical**  **singly charged  *m/z*** | **Experimental  singly charged  *m/z*** | **Mass error  (ppm)** |
| --- | --- | --- | --- | --- |
|  | [M + H] | 561.3754 | 561.3749 | 0. 9 |
| **1** | [M – H, OH] | 543.3648 | 543.3660 | -2.2 |
| **2** | [M – C_15_H_29_NO_5_] | 304.2124 | 304.2103 | 6.9 |
| **3** | [**2** – H, OH] | 286.2019 | 286.2025 | -2.1 |
| **4** | [**3** – H, OH] | 268.1913 | 268.1901 | 4.5 |
| **5** | [M – C_16_H_29_NO_4_] | 276.1811 | 276.1803 | 2.9 |
| **6** | [**5** – H, OH] | 258.1706 | 258.1696 | 3.9 |
| **7** | [**6** – H, OH] | 240.1600 | 240.1584 | 6.7 |
| **8** | [**8** – C=O] | 212.1650 | 212.1626 | 11.3 |
| **9** | [**7** – Ser] | 153.1279 | 153.1275 | 2.6 |
| **10** | [M – C_25_H_45_NO_6_] | 106.0504 | 106.0498 | 5.7 |
| **11** | [**4** – Ser] | 181.1592 | 181.1585 | 3.9 |

**Figure S7**. Fragmentation profile and structure of the compound ion [M+H]^+^ at *m/z* 571.3931.

**Table S8**. Summary of the theoretical and experimental fragmentation ions and mass error for the compound ion [M+H]^+^ at *m/z* 571.3931.

| **Proposed ion** | | **Theoretical**  **singly charged  *m/z*** | **Experimental  singly charged  *m/z*** | **Mass error  (ppm)** |
| --- | --- | --- | --- | --- |
|  | [M + H] | 571.3958 | 571.3931 | 5.0 |
| **1** | [M – H, OH] | 553.3853 | 553.3831 | 4.0 |
| **2** | [M – C=O – H, OH] | 525.3903 | 525.3911 | -1.5 |
| **3** | [M – C_15_H_29_NO_3_] | 304.2124 | 304.2106 | 5.9 |
| **4** | [**3** – H, OH] | 286.2019 | 286.2004 | 5.2 |
| **5** | [**4** – H, OH] | 268.1913 | 268.1897 | 5.0 |
| **6** | [**5** – C=O] | 240.1964 | 240.1944 | 8.3 |
| **7** | [**6** – H, OH] | 222.1858 | 222.1853 | 2.3 |

**Figure S8**. Fragmentation profile and structure of the compound ion [M+H]^+^ at *m/z* 559.3600.

**Table S9**. Summary of the theoretical and experimental fragmentation ions and mass error for the compound ion [M+H]^+^ at *m/z* 559.3600.

| **Proposed ion** | | **Theoretical**  **singly charged  *m/z*** | **Experimental  singly charged  *m/z*** | **Mass error  (ppm)** |
| --- | --- | --- | --- | --- |
|  | [M + H] | 559.3594 | 559.3600 | -1.0 |
| **1** | [M – H, OH] | 541.3489 | 541.3496 | -1.3 |
| **2** | [**1** – H, OH] | 523.3383 | 523.3380 | 0.6 |
| **3** | [M – Ser – H, OH] | 454.3168 | 454.3156 | 2.6 |
| **4** | [M – C_13_H_25_NO_4_] | 302.1967 | 302.1963 | 1.3 |
| **5** | [**4** – H, OH] | 284.1862 | 284.1851 | 3.9 |
| **6** | [**5** – H, OH] | 266.1756 | 266.1729 | 10.1 |
| **7** | [M – C_15_H_27_NO_4_] | 276.1811 | 276.1808 | 1.1 |
| **8** | [**7** – H, OH] | 258.1705 | 258.1701 | 1.6 |
| **9** | [**6** – Ser] | 179.1436 | 179.1419 | 9.5 |
| **10** | [M – C_25_H_45_NO_6_] | 106.0504 | 106.0497 | 6.6 |

**Figure S9**. Fragmentation profile and putative structure of the compound ion [M+H]^+^ at *m/z* 587.3908.

**Table S10**. Summary of the theoretical and experimental fragmentation ions and mass error for the compound ion [M+H]^+^ at *m/z* 587.3908.

| **Proposed ion** | | **Theoretical**  **singly charged  *m/z*** | **Experimental  singly charged  *m/z*** | **Mass error  (ppm)** |
| --- | --- | --- | --- | --- |
|  | [M + H] | 587.3907 | 587.3908 | -0.1 |
| **1** | [M – H, OH] | 569.3802 | 569.3787 | 2.6 |
| **2** | [M – C_15_H_29_NO_4_] | 302.1967 | 302.1951 | 5.3 |
| **3** | [**2** – H, OH] | 284.1862 | 284.1854 | 2.8 |
| **4** | [**3** – H, OH] | 266.1756 | 266.1751 | 1.9 |
| **5** | [M – C_15_H_27_NO_4_] | 304.2124 | 304.2101 | 7.6 |
| **6** | [**5** – H, OH] | 286.2018 | 286.2006 | 4.2 |
| **7** | [**6** – H, OH] | 268.1912 | 268.1924 | -4.5 |
| **8** | [**4** – Ser] | 179.1436 | 179.1463 | 0.0 |
| **9** | [**8** – H, OH] | 161.1330 | 161.1337 | -4.3 |
| **10** | [M – C_27_H_47_NO_6_] | 106.0504 | 106.0498 | 5.7 |

**Figure S10**. Fragmentation profile and putative structure of the compound ion [M+H]^+^ at *m/z* 585.3738.

**Table S11**. Summary of the theoretical and experimental fragmentation ions and mass error for the compound ion [M+H]^+^ at *m/z* 585.3738.

| **Proposed ion** | | **Theoretical**  **singly charged  *m/z*** | **Experimental  singly charged  *m/z*** | **Mass error  (ppm)** |
| --- | --- | --- | --- | --- |
|  | [M + H] | 585.3751 | 585.3738 | 2.3 |
| **1** | [M – H, OH] | 567.3646 | 567.3681 | -6.2 |
| **2** | [M – C_15_H_27_NO_4_] | 302.1967 | 302.1949 | 6.0 |
| **3** | [**2** – H, OH] | 284.1862 | 284.1844 | 6.3 |
| **4** | [**3** – H, OH] | 266.1756 | 266.1736 | 7.5 |
| **5** | [**4** – Ser] | 179.1436 | 179.1417 | 10.6 |
| **6** | [**5** – H, OH] | 161.1330 | 161.1326 | 2.5 |
| **7** | [M – C_27_H_45_NO_6_] | 106.0504 | 106.0491 | 12.3 |


**Figure S11**. Fragmentation profile and structure of the compound ion [M+H]^+^ at *m/z* 585.4117.



**Table S12**. Summary of the theoretical and experimental fragmentation ions and mass error for the compound ion [M+H]^+^ at *m/z* 585.4117.

| **Proposed ion** | | **Theoretical**  **singly charged  *m/z*** | **Experimental  singly charged  *m/z*** | **Mass error  (ppm)** |
| --- | --- | --- | --- | --- |
|  | [M + H] | 585.4115 | 585.4117 | -0.3 |
| **1** | [M – C_10_H_17_NO_5_] | 354.3008 | 354.2999 | 2.5 |
| **2** | [**1** + Na – H] | 376.2828 | 376.2832 | -1.1 |
| **3** | [M – C_21_H_39_NO_3_] | 232.1185 | 232.1163 | 9.5 |
| **4** | [**3** – H, OH] | 214.1079 | 214.1069 | 4.7 |
| **5** | [**4** – H, OH] | 196.0973 | 196.0971 | 1.0 |
| **6** | [**4** – C_4_H_6_O] | 144.0660 | 144.0654 | 4.2 |
| **7** | [**6** – H, OH] | 126.0555 | 126.0549 | 4.8 |
| **8** | [**5** – CH_2_O] | 166.0868 | 166.0854 | 8.4 |

**Figure S12**. Fragmentation profile and structure of the compound ion [M+H]^+^ at *m/z* 573.4136.

**Table S13**. Summary of the theoretical and experimental fragmentation ions and mass error for the compound ion [M+H]^+^ at *m/z* 573.4136.

| **Proposed ion** | | **Theoretical**  **singly charged  *m/z*** | **Experimental  singly charged  *m/z*** | **Mass error  (ppm)** |
| --- | --- | --- | --- | --- |
|  | [M] | 573.4115 | 573.4136 | -3.7 |
| **1** | [M - C_10_H_17_NO_5_] | 342.3008 | 342.2991 | 5.0 |
| **2** | [**1** + Na - H] | 364.2828 | 364.2823 | 1.4 |
| **3** | [M - C_20_H_39_NO_3_] | 232.1185 | 232.1186 | -0.4 |
| **4** | [**3** - H_2_O] | 214.1079 | 214.1080 | -0.5 |
| **5** | [**4** - H_2_O] | 196.0973 | 196.0967 | 3.1 |
| **6** | [**4** – C_4_H_6_O] | 144.0660 | 144.0646 | 9.7 |
| **7** | [**6** – H, OH] | 126.0555 | 126.0544 | 8.7 |
| **8** | [**5** – CH_2_O] | 166.0868 | 166.0862 | 3.6 |

**Figure S13**. Fragmentation profile and structure of the compound ion [M+H]^+^ at *m/z* 559.3953.

**Table S14**. Summary of the theoretical and experimental fragmentation ions and mass error for the compound ion [M+H]^+^ at *m/z* 559.3953.

| **Proposed ion** | | **Theoretical**  **singly charged  *m/z*** | **Experimental  singly charged  *m/z*** | **Mass error  (ppm)** |
| --- | --- | --- | --- | --- |
|  | [M + H] | 559.3958 | 559.3953 | 0.9 |
| **1** | [M – C_10_H_17_NO_5_] | 328.2851 | 328.2819 | 9.8 |
| **2** | [**1** + Na – H] | 350.2671 | 350.2667 | 1.1 |
| **3** | [M – C_19_H_37_NO_3_] | 232.1185 | 232.1181 | 1.7 |
| **4** | [**3** – H, OH] | 214.1079 | 214.1079 | 0.0 |
| **5** | [**4** – H, OH] | 196.0973 | 196.0969 | 2.0 |
| **6** | [**4** – C_4_H_6_O] | 144.0660 | 144.0661 | -0.7 |
| **7** | [**6** – H, OH] | 126.0555 | 126.0559 | -3.2 |
| **8** | [**5** – CH_2_O] | 166.0868 | 166.0868 | 0.0 |

**Figure S14**. Fragmentation profile and putative structure of the compound ion [M+H]^+^ at *m/z* 557.3804.

**Table S15**. Summary of the theoretical and experimental fragmentation ions and mass error for the compound ion [M+H]^+^ at *m/z* 557.3804.

| **Proposed ion** | | **Theoretical**  **singly charged  *m/z*** | **Experimental  singly charged  *m/z*** | **Mass error  (ppm)** |
| --- | --- | --- | --- | --- |
|  | [M + H] | 557.3802 | 557.3804 | -0.3 |
| **1** | [M – C_10_H_17_NO_5_] | 326.2695 | 326.2669 | 8.0 |
| **2** | [**1** + Na – H] | 348.2515 | 348.2509 | 1.7 |
| **3** | [M – C_19_H_35_NO_3_] | 232.1185 | 232.1177 | 3. 5 |
| **4** | [**3** – H, OH] | 214.1079 | 214.1082 | -1.4 |
| **5** | [**4** – H, OH] | 196.0973 | 196.0981 | -4.1 |
| **6** | [**4** – C_4_H_6_O] | 144.0660 | 144.0661 | -0.7 |
| **7** | [**6** – H, OH] | 126.0555 | 126.0546 | 7.1 |
| **8** | [**5** – CH_2_O] | 166.0868 | 166.0860 | 4.8 |

**Figure S15**. Fragmentation profile and putative structure of the compound ion [M+H]^+^ at *m/z* 589.4056.

**Table S16**. Summary of the theoretical and experimental fragmentation ions and mass error for the compound ion [M+H]^+^ at *m/z* 589.4056.

| **Proposed ion** | | **Theoretical**  **singly charged  *m/z*** | **Experimental  singly charged  *m/z*** | **Mass error  (ppm)** |
| --- | --- | --- | --- | --- |
|  | [M + H] | 589.4064 | 589.4056 | 1.4 |
| **1** | [M – C_10_H_17_NO_5_] | 358.2957 | 358.2958 | -0.2 |
| **2** | [**1** – H, OH] | 340.2851 | 340.2863 | -3.5 |
| **3** | [**1** + Na – H] | 380.2777 | 380.2774 | 0.8 |
| **4** | [M – C_20_H_39_NO_4_] | 232.1185 | 232.1190 | -2.2 |
| **5** | [**4** – H, OH] | 214.1079 | 214.1077 | 0.9 |
| **6** | [**5** – H, OH] | 196.0973 | 196.0966 | 3.6 |
| **7** | [**5** – C_4_H_6_O] | 144.0660 | 144.0658 | 1.4 |
| **8** | [**7** – H, OH] | 126.0555 | 126.0543 | 9.5 |
| **9** | [**6** – CH_2_O] | 166.0868 | 166.0854 | 8.4 |

**Figure S16**. Fragmentation profile and putative structure of the compound ion [M+H]^+^ at *m/z* 583.3947.

**Table S17**. Summary of the theoretical and experimental fragmentation ions and mass error for the compound ion [M+H]^+^ at *m/z* 583.3947.

| **Proposed ion** | | **Theoretical**  **singly charged  *m/z*** | **Experimental  singly charged  *m/z*** | **Mass error  (ppm)** |
| --- | --- | --- | --- | --- |
|  | [M + H] | 583.3958 | 583.3947 | 2.1 |
| **1** | [M – C_10_H_17_NO_5_] | 352.2851 | 352.2823 | 8.0 |
| **2** | [**1** + Na – H] | 374.2671 | 374.2656 | 4.0 |
| **3** | [M – C_21_H_37_NO_3_] | 232.1185 | 232.1186 | -0.4 |
| **4** | [**3** – H, OH] | 214.1079 | 214.1065 | 6.5 |
| **5** | [**4** – H, OH] | 196.0973 | 196.0966 | 3.6 |
| **6** | [**4** – C_4_H_6_O] | 144.0660 | 144.0647 | 9.0 |
| **7** | [**6** – H, OH] | 126.0555 | 126.0542 | 10.3 |
| **8** | [**5** – CH_2_O] | 166.0868 | 166.0854 | 8.4 |

**Figure S17**. Fragmentation profile and putative structure of the compound ion [M+H]^+^ at *m/z* 575.3902.

**Table S18**. Summary of the theoretical and experimental fragmentation ions and mass error for the compound ion [M+H]^+^ at *m/z* 575.3902.

| **Proposed ion** | | **Theoretical**  **singly charged  *m/z*** | **Experimental  singly charged  *m/z*** | **Mass error  (ppm)** |
| --- | --- | --- | --- | --- |
|  | [M + H] | 575.3907 | 575.3902 | 0.9 |
| **1** | [M – C_10_H_17_NO_5_] | 344.2801 | 344.2798 | 0.9 |
| **2** | [**1** + Na – H] | 366.2621 | 366.2610 | 3.0 |
| **3** | [M – C_19_H_37_NO_4_] | 232.1185 | 232.1168 | 7.3 |
| **4** | [**3** – H, OH] | 214.1079 | 214.1077 | 0.9 |
| **5** | [**4** – H, OH] | 196.0973 | 196.0954 | 9.7 |
| **6** | [**4** – C_4_H_6_O] | 144.0660 | 144.0653 | 4.9 |
| **7** | [**6** – H, OH] | 126.0555 | 126.0544 | 8.7 |
| **8** | [**5** – CH_2_O] | 166.0868 | 166.0856 | 7.2 |
| **9** | [**1** – H, OH] | 326.2695 | 326.2694 | 0.4 |

**Figure S18**. Fragmentation profile and putative structure of the compound ion [M+H]^+^ at *m/z* 545.3787.

**Table S19**. Summary of the theoretical and experimental fragmentation ions and mass error for the compound ion [M+H]^+^ at *m/z* 545.3787.

| **Proposed ion** | | **Theoretical**  **singly charged  *m/z*** | **Experimental  singly charged  *m/z*** | **Mass error  (ppm)** |
| --- | --- | --- | --- | --- |
|  | [M + H] | 545.3802 | 545.3787 | 2.8 |
| **1** | [M – C_10_H_17_NO_5_] | 314.2695 | 314.2716 | -6.7 |
| **2** | [**1** + Na – H] | 336.2515 | 336.2500 | 4.5 |
| **3** | [M – C_18_H_35_NO_3_] | 232.1185 | 232.1163 | 9.5 |
| **4** | [**3** – H, OH] | 214.1079 | 214.1071 | 3.7 |
| **5** | [**4** – H, OH] | 196.0973 | 196.0962 | 5.6 |
| **6** | [**4** – C_4_H_6_O] | 144.0660 | 144.0646 | 9.7 |
| **7** | [**6** – H, OH] | 126.0555 | 126.0550 | 4.0 |
| **8** | [**5** – CH_2_O] | 166.0868 | 166.0868 | 0.0 |

**Figure S19**. Fragmentation profile and putative structure of the compound ion [M+H]^+^ at *m/z* 599.4265.

**Table S20**. Summary of the theoretical and experimental fragmentation ions and mass error for the compound ion [M+H]^+^ at *m/z* 599.4265.

| **Proposed ion** | | **Theoretical**  **singly charged  *m/z*** | **Experimental  singly charged  *m/z*** | **Mass error  (ppm)** |
| --- | --- | --- | --- | --- |
|  | [M + H] | 599.4271 | 599.4265 | 1.0 |
| **1** | [M – C_10_H_17_NO_5_] | 368.3164 | 368.3160 | 1.1 |
| **2** | [**1** + Na – H] | 390.2984 | 390.2947 | 9.5 |
| **3** | [M – C_22_H_41_NO_3_] | 232.1185 | 232.1179 | 2.6 |
| **4** | [**3** – H, OH] | 214.1079 | 214.1068 | 5.1 |
| **5** | [**4** – H, OH] | 196.0973 | 196.0961 | 6.1 |
| **6** | [**4** – C_4_H_6_O] | 144.0660 | 144.0647 | 9.0 |
| **7** | [**6** – H, OH] | 126.0555 | 126.0548 | 5.6 |
| **8** | [**5** – CH_2_O] | 166.0868 | 166.0857 | 6.6 |


**Figure S20**. Fragmentation profile and putative structure of the compound ion [M+H]^+^ at *m/z* 587.4268.

**Table S21**. Summary of the theoretical and experimental fragmentation ions and mass error for the compound ion [M+H]^+^ at *m/z* 587.4268.

| **Proposed ion** | | **Theoretical**  **singly charged  *m/z*** | **Experimental  singly charged  *m/z*** | **Mass error  (ppm)** |
| --- | --- | --- | --- | --- |
|  | [M + H] | 587.4271 | 587.4268 | 0.6 |
| **1** | [M – C_10_H_17_NO_5_] | 356.3164 | 356.3164 | 0.0 |
| **2** | [**1** + Na – H] | 378.2984 | 378.2987 | -0.8 |
| **3** | [M – C_21_H_41_NO_3_] | 232.1185 | 232.1182 | 1.3 |
| **4** | [**3** – H, OH] | 214.1079 | 214.1074 | 2.3 |
| **5** | [**4** – H, OH] | 196.0973 | 196.0972 | 0.5 |
| **6** | [**4** – C_4_H_6_O] | 144.0660 | 144.0658 | 1.4 |
| **7** | [**6** – H, OH] | 126.0555 | 126.0550 | 4.0 |
| **8** | [**5** – CH_2_O] | 166.0868 | 166.0867 | 0.6 |

**Figure S21**. Fragmentation profile and putative structure of the compound ion [M+H]^+^ at *m/z* 627.4192.

**Table S22**. Summary of the theoretical and experimental fragmentation ions and mass error for the compound ion [M+H]^+^ at *m/z* 627.4192.

| **Proposed ion** | | **Theoretical**  **singly charged  *m/z*** | **Experimental  singly charged  *m/z*** | **Mass error  (ppm)** |
| --- | --- | --- | --- | --- |
|  | [M + H] | 627.4220 | 627.4192 | 4.5 |
| **1** | [M – C_12_H_19_NO_6_] | 354.3008 | 354.3016 | -2.3 |
| **2** | [**1** + Na – H] | 376.2828 | 376.2820 | 2.1 |
| **3** | [M – C_21_H_39_NO_3_] | 274.1290 | 274.1287 | 1.1 |
| **4** | [**3** – H, OH] | 256.1185 | 256.1174 | 4.3 |
| **5** | [**4** – C_4_H_6_O] | 144.0660 | 144.0663 | -2.1 |
| **6** | [**5** – H, OH] | 126.0555 | 126.0545 | 7.9 |

**Figure S22**. The UPLC profiles (left panel) and ESI-MS spectra (right panel) of the serratamolide homologues produced by P1 and/or NP1 strains (representatives from both strains). The UPLC retention time (min) and main ion in the peak is indicated for each of the purified compounds. The *m/z* value of the major ion (singly charged) in the main peak fraction is shown in the ESI-MS spectrum corresponding to each UPLC chromatogram. Compound numbers correlate with those in Table 1.

**Figure S23**. The UPLC profiles (left panel) and ESI-MS spectra (right panel) of the glucosamine derivative homologues produced by P1 and/or NP1 strains (representatives from both strains). The UPLC retention time (min) and main ion in the peak is indicated for each of the purified compounds. The *m/z* value of the major ion (singly charged) in the main peak fraction is shown in the ESI-MS spectrum corresponding to each UPLC chromatogram. Compound numbers correlate with those in Table 2.

**References**

Dwivedi, D., Jansen, R., Molinari, G., Nimtz, M., Johri, B.N., and Wray, V. (2008). Antimycobacterial serratamolides and diacyl peptoglucosamine derivatives from *Serratia* sp. J. Nat. Prod. 71:4, 637–641. https://doi.org/10.1021/np7007126

Eckelmann, D., Spiteller, M., and Kusari, S. (2018). Spatial-temporal profiling of prodiginines and serratamolides produced by endophytic *Serratia marcescens* harbored in *Maytenus serrata*. Sci. Rep. 8, 1–15. https://doi.org/[10.1038/s41598-018-23538-5](https://doi.org/10.1038/s41598-018-23538-5)

Seyedsayamdost, M.R., Cleto, S., Carr, G., Vlamakis, H., João Vieira, M., Kolter, R., and Clardy, J. (2012). Mixing and matching siderophore clusters: structure and biosynthesis of serratiochelins from *Serratia* sp. V4. J. Am. Chem. Soc. 134:33, 13550–13553. https://doi.org/10.1021/ja304941d
